# *Teosinte Pollen Drive* guides maize diversification and domestication by RNAi

**DOI:** 10.1038/s41586-024-07788-0

**Published:** 2024-08-07

**Authors:** Benjamin Berube, Evan Ernst, Jonathan Cahn, Benjamin Roche, Cristiane de Santis Alves, Jason Lynn, Armin Scheben, Daniel Grimanelli, Adam Siepel, Jeffrey Ross-Ibarra, Jerry Kermicle, Robert A. Martienssen

**Affiliations:** 1 Howard Hughes Medical Institute, Cold Spring Harbor Laboratory, Cold Spring Harbor, NY USA; 2 Simons Center for Quantitative Biology, Cold Spring Harbor Laboratory, Cold Spring Harbor, NY USA; 3 DIADE, Université de Montpellier, Montpellier, France; 4 Department of Evolution and Ecology, Center for Population Biology and Genome Center, University of California at Davis, Davis, CA USA; 5 Laboratory of Genetics, University of Wisconsin, Madison, WI USA

**Keywords:** Genetic hybridization, Plant domestication, RNAi, Evolutionary genetics, Plant genetics

## Abstract

Selfish genetic elements contribute to hybrid incompatibility and bias or ‘drive’ their own transmission^1,2^. Chromosomal drive typically functions in asymmetric female meiosis, whereas gene drive is normally post-meiotic and typically found in males. Here, using single-molecule and single-pollen genome sequencing, we describe *Teosinte Pollen Drive*, an instance of gene drive in hybrids between maize (*Zea mays* ssp. *mays*) and teosinte *mexicana* (*Z. mays* ssp. *mexicana*) that depends on RNA interference (RNAi). 22-nucleotide small RNAs from a non-coding RNA hairpin in *mexicana* depend on *Dicer-like 2* (*Dcl2*) and target *Teosinte Drive Responder 1* (*Tdr1*), which encodes a lipase required for pollen viability. *Dcl2*, *Tdr1* and the hairpin are in tight pseudolinkage on chromosome 5, but only when transmitted through the male. Introgression of *mexicana* into early cultivated maize is thought to have been critical to its geographical dispersal throughout the Americas^3^, and a tightly linked inversion in *mexicana* spans a major domestication sweep in modern maize^4^. A survey of maize traditional varieties and sympatric populations of teosinte *mexicana* reveals correlated patterns of admixture among unlinked genes required for RNAi on at least four chromosomes that are also subject to gene drive in pollen from synthetic hybrids. *Teosinte Pollen Drive* probably had a major role in maize domestication and diversification, and offers an explanation for the widespread abundance of ‘self’ small RNAs in the germ lines of plants and animals.

## Abstract (web summary)

An example of hybrid incompatibility between maize and teosinte reveals a selfish toxin–antidote system mediated by small RNAs that may have contributed to the origin of maize.

## Main

The introduction of novel genetic variation through hybridization is an important evolutionary catalyst^5^, as adaptive introgression in hybrid individuals can increase fitness under new environmental conditions and lead to geographical expansion and diversification^6^. Modern maize, for example, was first domesticated from a close relative of *Z. mays* ssp. *parviglumis* (teosinte *parviglumis*) in the lowlands of southwest Mexico approximately 9000 bp, but admixture from a second teosinte, *Z. mays* ssp. *mexicana*, 4,000 years later, appears to have catalysed rapid expansion across the Americas^3^. The combination of divergent genomes, however, can also result in hybrid sterility, inviability and necrosis^7–9^. The Bateson–Dobzhansky–Muller (BDM) model accounts for such scenarios, via the interaction of deleterious mutations in distinct populations and at least some of these incompatibilities stem from intragenomic conflict triggered by selfish genetic elements^2,10^.

Meiotic drive depends on selfish elements that actively manipulate reproductive development to facilitate their own preferential transmission^11^. Chromosomal drive refers to the manipulation of chromosome segregation during asymmetric female meiosis, as centromeres, heterochromatic knobs and telomeres exert mechanical advantages that favour their inclusion in the egg cell^1,12–14^. Examples include *Abnormal 10* (*Ab10*) in both maize and teosinte populations^15,16^. Conversely, gene drive occurs preferentially in males and is achieved via disruption of post-meiotic reproductive development resulting in segregation distortion^17,18^. These systems tend to occur in sperm or haploid spores and involve toxin–antidote (or distorter–responder) pairs in close genetic linkage. Gametes that do not inherit the drive locus are selectively killed, resulting in overrepresentation of the driver^11^. The mouse *t*-complex^19,20^, *Drosophila Segregation Distorter* (*SD*) complex^21,22^ and *Schizosaccharomyces pombe/kombucha wtf* spore killers^23,24^ are all autosomal drivers that selectively kill competing wild-type gametes in heterozygotes.

Because selfish genetic elements often impose fitness and fertility penalties, tremendous selective pressure is placed on regions of the genome that can evolve suppressors^25^. As a consequence, drive systems undergo recurrent cycles of suppression and counter-suppression; although drive is predicted to be widespread, most systems exist in a cryptic state, either through suppression or fixation^11,26^. It is through hybridization with naive individuals that suppression is lost and drive is once again apparent^27^, reinforcing species barriers and influencing patterns of introgression in hybrid individuals via genetic linkage^28,29^.

Here we characterize a male-specific segregation distortion system in introgression lines between maize (*Z. mays* ssp. *Mays*) and teosinte *mexicana* (*Z. mays* ssp. *mexicana*), hereafter referred to as *Teosinte Pollen Drive* (*TPD*). We implicate small interfering RNAs (siRNAs) from a *mexicana-*specific long non-coding hairpin RNA in close genetic linkage with the centromere of chromosome 5 as the primary factor mediating pollen killing. Co-segregation of a genetically linked hypomorphic (partially functional) *Dcl2* allele suppresses this effect via the reduction of secondary 22-nucleotide (nt) siRNAs and is reinforced by a second unlinked antidote (*Tpd2*) on chromosome 6. Survey sequencing of modern and traditional varieties of maize from Mexico and sympatric populations of teosinte implicate *TPD* in patterns of *mexicana* introgression, and in maize dispersal and domestication.

## *TPD* in maize hybrids

**Fig. 1:**
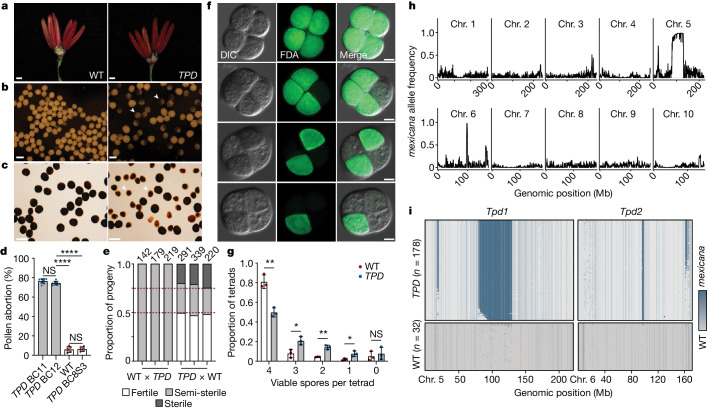
Single-pollen sequencing reveals selfish inheritance in *TPD*. **a**, Anther florets (5 mm) from wild-type (WT; left) and *TPD* (right) plants. Scale bars, 1 mm. **b**, Mature pollen grains from WT (left) and *TPD* (right) plants. Arrowheads denote developmentally arrested pollen grains. Scale bars, 0.1 mm. **c**, Viable pollen grains are plump and darkly stained with iodine potassium iodide (I_2_KI), whereas arrested pollen grains (arrowheads) exhibit reduced diameter and incomplete staining. Scale bars, 0.1 mm. **d**, Quantification of pollen abortion rates in *TPD* backcross (BC_11,12_), WT and *TPD* self-fertilized (BC_8_S_3_) lines. Data are mean ± s.d. (*n* = 6–8). ****P < 0.0001 and not significant (NS; two-tailed Student’s *t*-test). **e**, Phenotypic segregation ratios in replicate reciprocal crosses. The numbers above the bar represent the sample size for each progeny population. The red dashed lines denote a perfect 2:1:1 phenotypic segregation ratio. **f**, Fluorescein diacetate (FDA) viability staining of tetrads from *TPD* plants. Pollen viability is progressively restricted to a single spore following meiosis. Panels show differential interference contrast (DIC), FDA and merged images. Scale bars, 50 µm. **g**, Viability scoring of *TPD* and WT tetrads shown in panel **f**. *TPD* spores exhibit significantly reduced viability at the tetrad stage. *n* = 3 biological replicates, 952 total tetrads assayed. Data are mean ± s.d. *P < 0.05 and **P < 0.01 (Welch’s *t*-test). **h**, Single-pollen grain genome sequencing. Imputed allele frequencies at *mexicana* markers in a population of 178 mature pollen grains collected from *TPD* plants. Chr. chromosome. **i**, Imputed *mexicana* marker density on chromosomes 5 and 6 for individual pollen grain genome sequences. Multiple *mexicana* haplotypes (blue) are selfishly inherited in viable *TPD* pollen grains (*n* = 178) but not in WT pollen grains (*n* = 32). Values shown are plotted using a 500-kb sliding window (**h**,**i**).

**Extended Data Fig. 1:**
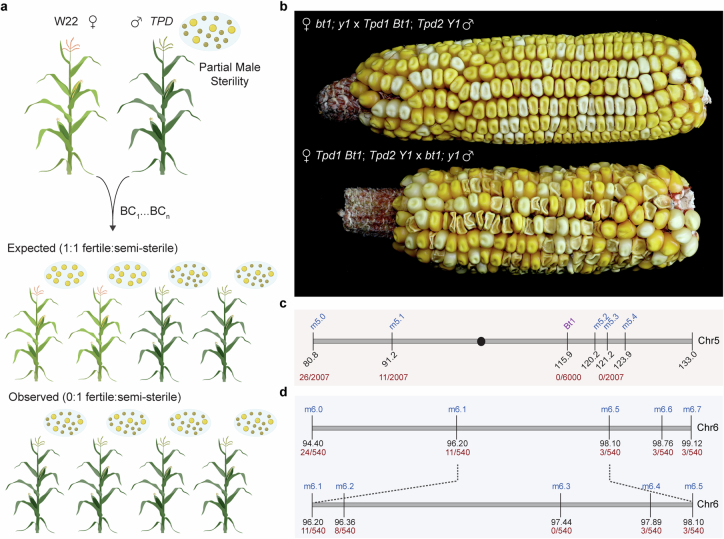
*Teosinte Pollen Drive* and genetic mapping of *Tpd1* and *Tpd2.* **a**, Crossing scheme of the *TPD* phenotype. When back-crossed as male, all the progeny of semi-sterile *TPD* plants display the semi-sterile pollen phenotype instead of the expected 1:1 fertile:semi-sterile ratio. Graphics in panel **a** were created using BioRender (https://biorender.com). **b**, Representative ears from *Tpd1 Bt1/bt1*; *Tpd2 Y1/y1* reciprocal crosses with *bt1; y1* testers, demonstrating severe segregation distortion (“drive” of *Bt1*), but only through the male. *bt1 (brittle1*, collapsed kernels); *y1* (*yellow1*, white kernels). **c**, Summary of molecular and morphological mapping of the *Tpd1* interval. Molecular mapping was performed using *Tpd/++* x W22 segregating progeny, whereas morphological mapping was performed by crossing *Tpd1 Bt1/tpd1 bt1* plants to *bt1* testers. **d**, Molecular mapping of the *Tpd2* interval. SNP markers are shown in blue with recombination frequencies in red.

Hybridization between maize and teosinte is subject to unilateral cross-incompatibility^30,31^, but pollination of maize by *mexicana* pollen is frequent^32^. Consistently, genome-wide assessments of introgression in sympatric collections have provided evidence for asymmetric gene flow from *mexicana* to maize^32,33^. To further explore the reproductive consequences of hybridization, multiple sympatric collections of *mexicana* were crossed to the Midwestern US dent inbred W22, resulting in variable rates of pollen abortion that typically decreased in subsequent generations. However, a subset of late backcross (BC) lines (hereafter *TPD*) displayed an unusually consistent rate of pollen abortion (75.5 ± 2.48%) relative to W22 (6.02 ± 2.95%; *P* < 0.0001, Welch’s *t*-test) despite normal vegetative and reproductive development (Fig. 1a–c and Extended Data Fig. 1a). The pollen abortion phenotype was absent after three rounds of selfing in *TPD* BC_8_S_3_ plants (6.40 ± 2.26%; *P* < 0.0001, Welch’s *t*-test), suggesting that heterozygosity was required (Fig. 1d). In reciprocal crosses, pollination of *TPD* ears with W22 pollen resulted in the independent assortment of fertile, semi-sterile and fully male sterile progeny in a 2:1:1 ratio (Fig. 1e and Supplementary Table 1). These results indicated the presence of two unlinked loci responsible for pollen survival that were transmitted to all individuals in the next generation, but only through pollen. Because this phenotype was observed only in heterozygotes, we reasoned that it stemmed from an incompatibility between the W22 genome and regions of *mexicana* introgression after meiosis, reminiscent of genic drivers that distort patterns of inheritance via selective gamete killing^20,24^. Consistently, meiotic progression in *TPD* plants was normal until the tetrad stage, following the separation of each haploid complement (Fig. 1f). This phenotype, although strictly post-meiotic, appeared to progress gradually, ultimately resulting in arrested pollen grains with a heterogenous overall diameter and varying degrees of starch accumulation (Fig. 1c,g).

Genetic mapping revealed that *brittle endosperm 1* (*bt1*) on chromosome 5 and *yellow endosperm 1* (*y1*) on chromosome 6 were linked with the pollen abortion phenotype (Extended Data Fig. 1b,c). Backcrosses to *y1*;*bt1* yielded 100% *Bt1* kernels instead of 50%, but only when *TPD* was used as a pollen parent (Extended Data Fig. 1b). The frequency of white kernels (*y1*) was in agreement with recombination estimates (21–22%). This bias was strongly indicative of gene drive resembling similar incompatibility systems in rice^34^, although we could not formally exclude other forms of incompatibility that also result in segregation distortion. To exclude such possibilities, we sequenced the genomes of two homozygous *TPD* lines (BC_8_S_3_ and BC_5_S_2_) to define 408,031 high-confidence single-nucleotide polymorphisms (SNPs) corresponding to regions of *mexicana* introgression. Next, we sequenced the genomes of individual surviving pollen grains from *TPD* plants, rationalizing that if segregation distortion was occurring in pollen, the causative regions would be overrepresented. We found that several intervals occurred at much higher frequencies than expected after eight backcrosses (Fig. 1h). Of note, introgression intervals on chromosomes 5 and 6 were consistently observed in all surviving pollen (Fig. 1i), strongly indicative of post-meiotic gene drive. We designated these loci as *Tpd1* and *Tpd2*, respectively.

## A *Dicer-Like 2* toxin–antidote complex

**Fig. 2:**
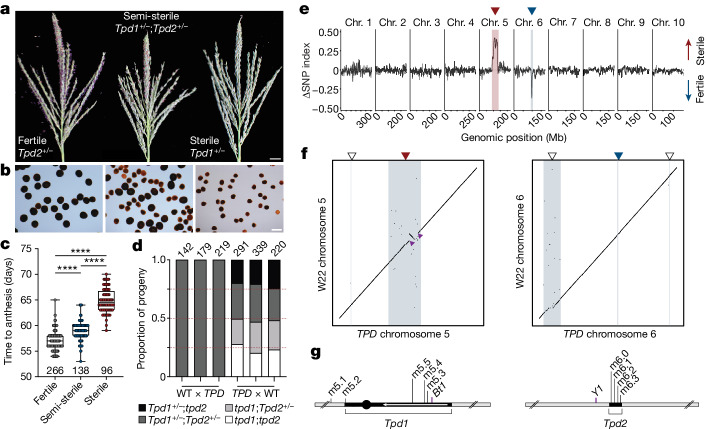
A toxin–antidote system introduced from *mexicana* on chromosomes 5 and 6. **a**, Representative tassels from fertile, semi-sterile and sterile plants in a maternally segregating population. Scale bar, 1 cm. **b**, I_2_KI viability staining of pollen from the same genotypes as in panel **a**. Scale bar, 0.1 mm. **c**, Measurement of days to anthesis in fertile, semi-sterile and sterile phenotypic classes. *n* is given at the bottom of the plots. ****P < 0.0001 (two-tailed Mann–Whitney test). **d**, Genotypic segregation ratios in reciprocal crosses. The numbers at the top of the bars represent the sample size for each progeny population. The red dashed lines denote a perfect 1:1:1:1 genotypic segregation ratio. Normal segregation is only observed in maternal progeny. **e**, Bulk segr**e**gant analysis of fertile and sterile progeny pools indicates that *Tpd1* (red arrowhead) is necessary and sufficient for dominant male sterility (toxin), whereas *Tpd2* (blue arrowhead) is associated with fertility (antidote). FDR ≤ 0.01 (Benjamini–Hochberg method). **f**, Dot plots of chromosomes 5 and 6 showing multiple alignment between the *TPD* and W22 reference genomes. The blue lines and shaded regions correspond to five fully scaffolded intervals of *mexicana* introgression (indicated by arrowheads). As in panel **e**, the red and blue arrowheads mark the *Tpd1* and *Tpd2* intervals, respectively. The small purple arrowheads indicate breakpoints of an approximately 13-Mb paracentric inversion present within the *Tpd1* haplotype on chromosome 5L. **g**, Schematics summarizing the *Tpd1* and *Tpd2* intervals, as well as associated markers. The 13-Mb inversion is indicated as a reverse arrow.

To determine the relative contributions of *Tpd1* and *Tpd2* to pollen abortion and survival, we separated the components by maternal transmission into fertile, semi-sterile (‘drive’) and fully sterile classes (Fig. 2a). Each progeny class had distinct rates of pollen abortion (Fig. 2b) and showed significant differences in flowering time (Fig. 2c). Fertile segregants were phenotypically wild type and showed no transmission defects, whereas drive plants recapitulated the canonical *TPD* pollen abortion phenotype. By contrast, male reproductive development in sterile plants was developmentally retarded, displaying severely delayed anthesis and reduced overall shed (Fig. 2a,c). Consequently, crosses performed with this pollen showed minimal seed set and often failed entirely. We collected pools of plants from the fertile and sterile phenotypic classes (Fig. 2d) for bulk segregant analysis, and found that *Tpd1* was differentially enriched in sterile plants, whereas *Tpd2* was enriched in fertile plants (Fig. 2e). This indicated that *Tpd1* alone was sufficient to ‘poison’ the male germ line and that this most likely occurred pre-meiotically, as only a single copy of *Tpd1* was required. Genetic mapping placed *Tpd1* in a large interval surrounding the centromere of chromosome 5, whereas *Tpd2* was placed in a 1.5-Mb interval on chromosome 6L (Extended Data Fig. 1c,d).

The unusual transmission of *TPD* led us to liken it to previously described selfish genetic elements that operate via post-meiotic gamete killing^20,22,24^. These systems generally encode a toxin (or distorter) that acts in *trans* to disrupt proper reproductive development. Only gametes containing a cell-autonomous antidote (or resistant responder allele) can suppress these effects in a gametophytic manner. Although the toxin was clearly encoded by *Tpd1*, the *TPD* system was unusual in that it featured a genetically unlinked antidote, namely, *Tpd2*. However, the absence of *tpd1;Tpd2* recombinants in the progeny of W22 × *TPD* crosses argued that *Tpd2* alone was insufficient for suppression of pollen abortion (Fig. 2d and Supplementary Table 2). We reasoned that this might reflect the additional requirement for another antidote, linked to *Tpd1*, that could explain the observed rate of pollen abortion (approximately 75%). Linked modifiers in drive systems are common and generally ascribed to the co-evolutionary struggle between distorters and rapidly accumulating suppressors^11,22^.

**Extended Data Fig. 2:**
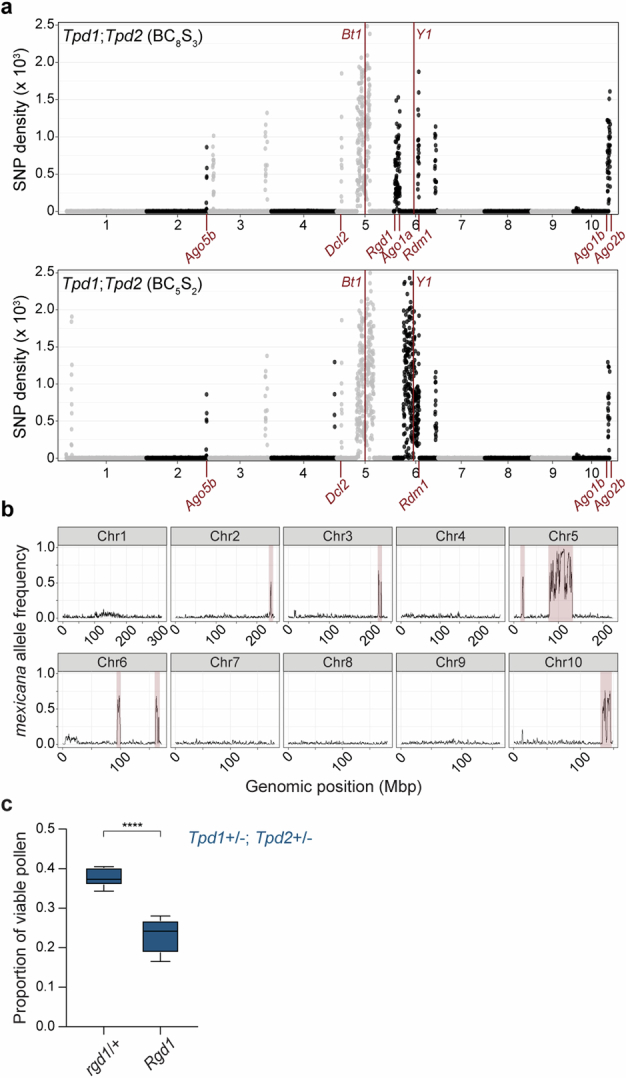
*mexicana* intervals introgressed into maize carry RNAi genes. **a**, Whole genome plots of homozygous *mexicana* SNP density present within *Tpd1; Tpd2* lines. The upper plot corresponds to data from bulked seedlings after 8 backcrosses and 3 self-pollinations (BC_8_S_3_) whereas the lower plot is from BC_5_S_2_ plants. SNP density is consolidated in 250 kb genomic bins. Physical locations for morphological markers *Bt1* and *Y1*, as well as *mexicana* derived RNAi genes, are labelled in red. 7/13 introgression intervals overlap in both independently maintained homozygous lines. **b**, Allele frequency at *mexicana* markers in 96 pollen grains from four different *TPD* plants subjected to single pollen grain sequencing. Regions highlighted in red were over-represented in viable pollen grains. **c**, Quantification of pollen viability in *Tpd1* + */−; Tpd2* + */−; rgd1/+* and *Tpd1* + */−; Tpd2* + */−; Rgd1* pollen demonstrating gametophytic suppression via germline segregation of the *rgd1* null allele. n ≥ 9 plants per genotype, ≥ 200 pollen grains per plant. **** p < 0.0001 (Welch’s t-test).

SNP genotyping of the two homozygous lines identified 13 *mexicana* introgression intervals, 7 of which were shared between backcross generations (Extended Data Fig. 2a). As predicted from the single-pollen sequencing data, the highest regions of SNP density were present on chromosome 5 (*Tpd1*) and chromosome 6 (*Tpd2*), coinciding with *Bt1* and close to *Y1*, respectively (Extended Data Fig. 2a). However, other regions strongly overrepresented in homozygous progeny were only partially overrepresented in *TPD* pollen, including additional peaks on chromosomes 5S, 6S and 6L (Extended Data Fig. 2b). This probably reflected the presence of recombinant pollen grains that competed poorly during pollination.

**Fig. 3:**
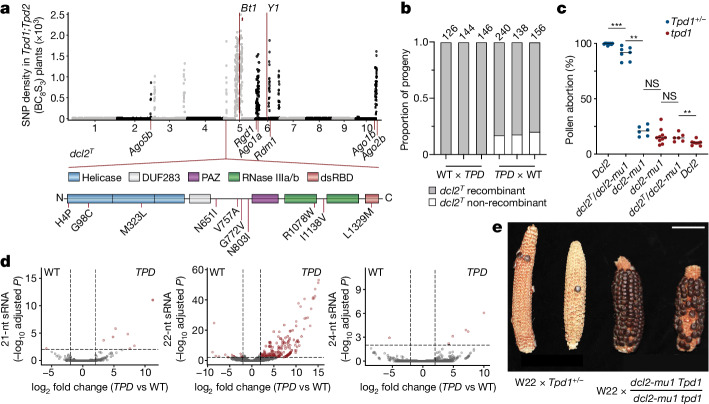
*Dcl2* from teosinte is a linked antidote for toxic 22-nt siRNA. **a**, Genome-wide *mexicana* SNP density in bulk-sequenced *Tpd1;Tpd2* (BC_8_S_3_) plants. A subset of *mexicana* introgression intervals (in addition to *Tpd1* and *Tpd2*) are selectively maintained and include RNAi factors. A *mexicana*-derived allele of *Dcl2* (*dcl2*^*T*^) with a high rate of nonsynonymous substitution is maintained in linkage to *Tpd1*. dsRBD, double-stranded RNA-binding domain. **b**, Rates of recombination between *dcl2*^*T*^ and *Tpd1* in replicate reciprocal crosses. *dcl2*^*T*^ exhibits tight pseudolinkage with *Tpd1* when propagated as male (0 cM), but not as female (18.7 ± 1.6 cM). The numbers above the bars represent the sample size for each progeny population. **c**, Measurements of pollen viability in *Tpd1/tpd1* and *tpd1* plants containing combinations of *Dcl2*, *dcl2*^*T*^ and *dcl2-mu1*. Addition of the *dcl2-mu1* hypomorphic allele is sufficient for suppression of *Tpd1*-mediated pollen abortion. Data points correspond to measurements from individual plants (*n* = 6–10). **P < 0.01 and ***P < 0.001 (two-tailed Mann–Whitney test). **d**, Volcano plots of 21-nt (*n* = 9), 22-nt (*n* = 212) and 24-nt (*n* = 6) small RNA (sRNA) clusters that are differentially expressed in WT and *TPD* pollen. The accumulation of ectopic 22-nt siRNAs occurs specifically in *TPD* pollen. log_2_ fold change ≥ 2, FDR ≤ 0.01. **e**, Representative ears from replicate crosses containing WT *Dcl2* (W22 × *Tpd1/tpd1*) or *dcl2-mu1* (W22 × *dcl2-mu1* *Tpd1/dcl2-mu1* tpd1) in linkage to *Tpd1*. Pollen parents homozygous for *dcl2-mu1* restore the seed set. Scale bar, 4 cm.

To determine gene content in these and other introgression intervals, we performed de novo genome assembly from homozygous *Tpd1;Tpd2* BC_8_S_3_ seedlings (see Methods; Supplementary Table 3) with fully scaffolded *mexicana* introgression intervals on chromosomes 5 and 6 (Fig. 2f,g). We noted the presence of a 1.9-Mb *mexicana* introgression interval on chromosome 5S linked to the *Tpd1* haplotype and strongly overrepresented in both our bulk sequencing and single-pollen grain data (Figs. 1h,i and 2f). Within this interval, we identified ten genes with expression in pollen, one of which, *Dcl2*, had excess nonsynonymous substitutions within conserved domains (Fig. 3a), suggesting the possibility of adaptive evolutionary change^35^. Absolute genetic linkage (*n* = 214) between this locus (hereafter *dcl2*^*T*^) and *Tpd1* was conditioned on passage through the male germ line from heterozygous *TPD* plants, whereas recombination between *dcl2*^*T*^ and *Tpd1* occurred at the expected frequency (approximately 12%) when crossed as female (Fig. 3b). This was very strong evidence for a linked antidote and probably explained the maintenance of this interval across 13 backcross generations.

*Dcl2* encodes a Dicer-like protein responsible for the production of 22-nt siRNAs from hairpins, as well as secondary small RNAs from double-stranded RNA templates produced by the coordinated action of RNA-DEPENDENT RNA POLYMERASE 6 (RDR6) and SUPPRESSOR OF GENE SILENCING 3 (SGS3)^36^. In *Arabidopsis thaliana*, DCL2 function is superseded by DCL4 and endogenous levels of 22-nt siRNAs are low^37^. However, DCL2 can fulfill roles in silencing and antiviral immunity when DCL4 function is lost^37,38^, sometimes resulting in ‘toxic’ pleiotropic defects associated with gene targets of 22-nt siRNAs^37,39,40^. These observations stem from the unique biological properties of 22-nt siRNAs, which are responsible for propagation of systemic silencing signals that move between cells^41^ and transitive amplification of silencing in both *cis* and *trans*^42^. In *dcl2*^*T*^, nonsynonymous changes were clustered within the DExD/H RNA helicase domain of Dicer (Fig. 3a), which has been shown to alter substrate preference and processing efficiency of double-stranded RNA, but not hairpin RNA, in both plants and invertebrates^43–45^.

To explore the role of 22-nt siRNAs in the *TPD* phenotype, we tested mutants in 22-nt siRNA biogenesis for their ability to act as antidotes. We isolated maternal *dcl2*^*T*^ recombinants and compared them with the *dcl2-mu1* allele in the W22 inbred background, which has a *Mu* transposon insertion in the 5′ untranslated region, 200 bp upstream of the start codon. In *dcl2*^*T*^/*dcl2-mu1* *Tpd1*, pollen abortion was partially suppressed, whereas pollen from *dcl2-mu1/dcl2-mu1* *Tpd1* plants were almost fully viable (Fig. 3c). This meant that stacking over the *dcl2*^*T*^ allele had a synergistic effect, strongly supporting its role as a partial antidote, and indicating that the sporophytic production of 22-nt siRNAs in diploid meiotic cells was responsible for the *TPD* phenotype. To test the idea that 22-nt siRNAs might be responsible for *TPD*, we sequenced pollen small RNAs from *TPD* and wild-type siblings and found that although small RNA composition was similar overall, the *Tpd1* haplotype triggered a strong, 22-nt-specific response (Fig. 3d). Consistent with these 22-nt small RNAs being responsible for the *TPD* phenotype, we observed almost complete rescue of sterility in *dcl2-mu1/dcl2-mu1* *Tpd1/* + pollen parents (Fig. 3e). Several other introgression intervals observed in one or the other backcross individual also included genes encoding components of the small RNA biogenesis pathway, including *ago1a*, *ago1b* and *rgd1*, the homologue of SGS3 (Fig. 3a and Extended Data Fig. 2a). These intervals were also observed in single-pollen grain sequencing along with *dcl2*^*T*^ (Extended Data Fig. 2b). To determine whether these genes were also capable of acting as an antidote, we crossed mutants in *rgd1* to *TPD* plants. Segregation of *rgd1* in the germ line of heterozygotes resulted in close to 50% viable pollen (Extended Data Fig. 2c), suggesting that it functions as a cell-autonomous gametophytic suppressor in a manner similar to *Tpd2*. We concluded that mutants in primary 22-nt small RNA synthesis (*dcl2-mu1*) blocked production of the toxin, whereas mutants in secondary 22-nt small RNA synthesis (*dcl2*^*T*^ and *rgd1*), and potentially in small RNA function (*ago1a* and *ago1b*), acted as antidotes.

## 22-nt small RNAs target a pollen lipase

**Extended Data Fig. 3:**
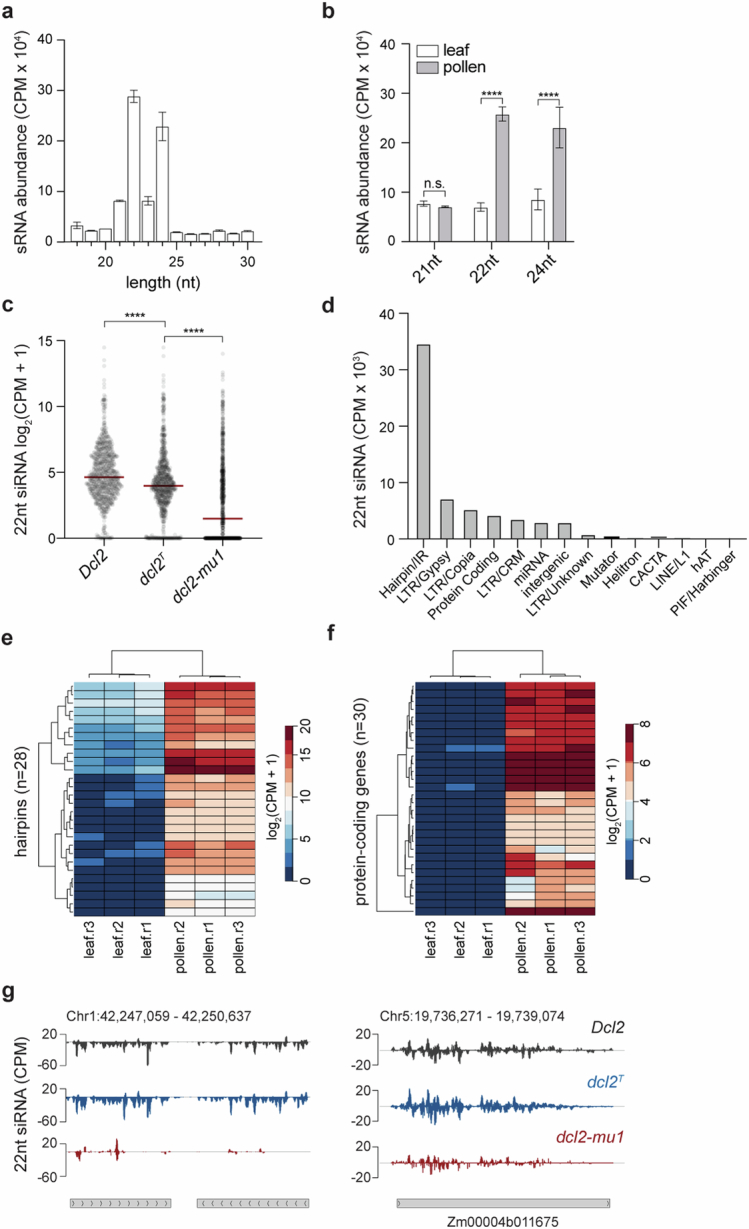
*DCL2*-dependent 22nt siRNAs from hairpins are prevalent in maize pollen. **a**, Distribution and relative abundances of small RNA size classes in WT pollen libraries. Bars indicate mean ± SD. n = 3 biological replicates. **b**, Comparison of relative abundances for 21-nt, 22-nt, and 24-nt sRNA size classes in WT leaf and pollen samples. Both 22-nt and 24-nt sRNAs show significant increases in pollen. Bars indicate mean ± SD. n = 3 biological replicates. **** p < 0.0001 (Welch’s t-test). **c**, Comparison of 22-nt sRNA levels in *Dcl2*, *dcl2*^*T*^, and *dcl2-mu1* pollen at 804 pollen-specific loci. Values shown are log_2_ transformed counts per million (CPM) averaged across replicates. n = 3 replicates per genotype. **** p < 0.0001 (ANOVA test). **d**, Summary of relative contributions for 22-nt sRNA producing loci in WT pollen. Hairpin/inverted repeat (IR) hp-siRNAs represent the largest fraction of 22-nt species. **e**, Heatmap showing 22-nt hp-siRNA levels at hpRNA loci in leaf and pollen. **f**, Heatmap showing 22-nt siRNA levels at protein-coding genes in leaf and pollen. **g**, Browser shots showing 22-nt hp-siRNA accumulation at a hpRNA locus on chromosome 1 (left) and 22 nt siRNA silencing at a representative protein-coding gene. Scale is CPM.

**Extended Data Fig. 4:**
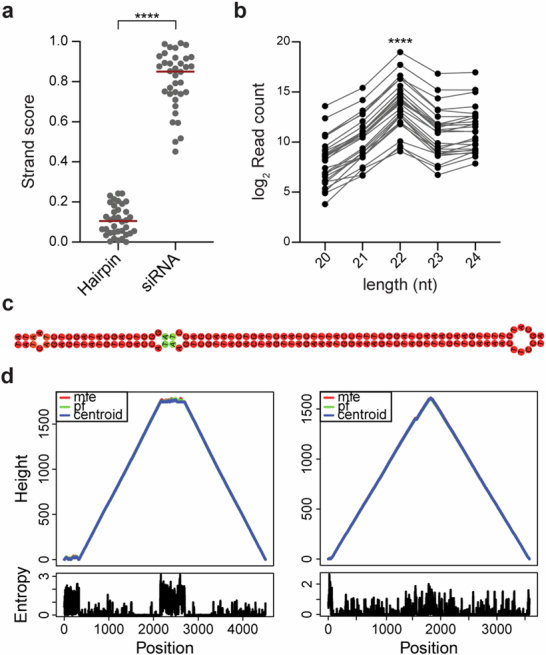
Validation of highly abundant pollen hairpin precursors. **a**, hp-siRNAs are expected to show strand bias. Measurement of strand score (min[plus, minus]/max[plus, minus]) at 28 putative hairpin precursors and randomly selected siRNA clusters from wild-type (W22) maize. A value of zero indicates complete strand bias, whereas a value of 1 indicates unbiased accumulation from both strands. n = 28. **** p < 0.0001 (Welch’s t-test). **b**, log2 read count at hairpin precursors indicate 22 nt size bias. n = 28. **** p < 0.0001 (ANOVA). **c**, Example of a 73 nt stretch from a 4,480 bp hairpin precursor demonstrating near-complete reverse complementarity. **d**, Mountain plots measuring thermodynamic stability of the *Tpd1* hairpin from *mexicana* and another randomly selected hairpin structure.

To identify the origin and the targets of DCL2-dependent small RNAs, we performed small RNA sequencing from wild-type, *dcl2*^*T*^ and *dcl2-mu1* plants. Analysis revealed that 22-nt siRNAs were the dominant species in wild-type pollen (Extended Data Fig. 3a,b) and defined 804 high-confidence 22-nt siRNA pollen-specific clusters (log_2_ fold change ≥ 2, false discovery rate (FDR) ≤ 0.01; Supplementary Table 4). As expected, these clusters depended on *Dcl2* (*P* < 0.0001, determined by analysis of variance (ANOVA)) and there were even fewer 22-nt siRNAs in *dcl2-mu1* than in *dcl2*^*T*^ (Extended Data Fig. 3c). Over half (54.6%) of all pollen-specific 22-nt species were derived from endogenous hairpin precursors (hpRNAs; Extended Data Fig. 3d,e,g). Hairpin short interfering RNAs (hp-siRNAs) were disproportionately 22 nt long, derived from a single strand (Extended Data Fig. 4a,b) with high thermodynamic stability (Extended Data Fig. 4c,d). On the basis of these criteria (and a minimum expression cut-off), we identified 28 hp-siRNA-producing loci in the genome, with at least one hairpin on every chromosome except chromosome 4 (average 2.1 ± 1.3 per chromosome). hp-siRNAs can serve as a powerful means to silence transposons^46^, and 22-nt siRNAs targeting *Gypsy* and *Copia* LTR retrotransposons were abundant in pollen, as were those targeting *Mutator* and *CACTA* elements (Extended Data Fig. 3d). We also found evidence for pollen-specific silencing of at least 30 protein-coding genes (Extended Data Fig. 3d,f,g). Germline specificity is a common feature in *SD* systems, as such factors can avoid the evolutionary conflicts imposed by pleiotropic fitness defects in the diploid stage of the life cycle^47^.

**Extended Data Fig. 5:**
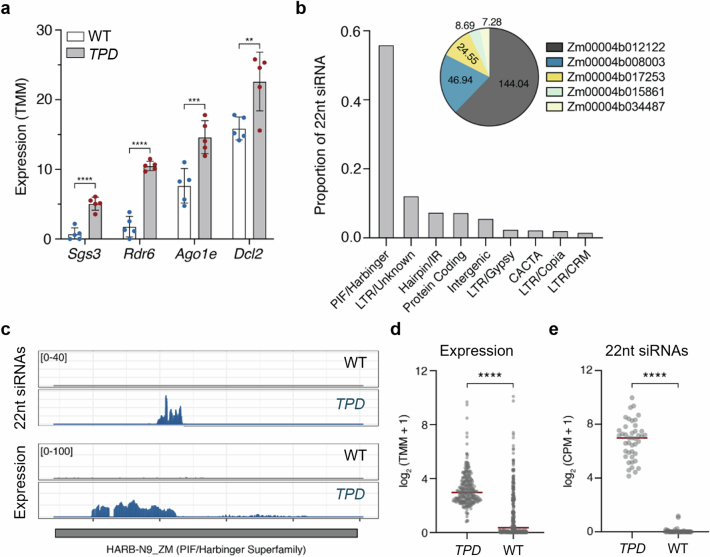
Origins and targets of 22 nt small RNAs in *TPD* Pollen. **a**, RNAi genes (*Sgs3*/*Rgd1*, *Rdr6*, *Ago1e* and *Dcl2*) associated with 22 nt biogenesis and function are upregulated in *TPD* pollen. Expression is shown in TMM normalized counts. Bars show mean ± SD. n = 5 replicates per condition. **** p < 0.0001, *** p < 0.001, ** p < 0.01 (FDR). **b**, Relative abundances of *TPD*-dependent 22 nt siRNAs mapping to annotated elements. Pie chart inset shows proportions of 22 nt siRNAs targeting genes in CPM. **c**, Browser shot showing transcriptional activation at PIF/Harbinger elements in *TPD* pollen as well as 22 nt siRNA accumulation. **d**, Quantification of mRNA expression at 258 PIF/Harbinger superfamily elements in WT and *TPD* pollen. **** p < 0.0001 (Mann-Whitney test). **e**, 22 nt siRNA levels at 42 PIF/Harbinger elements in WT and *TPD* pollen. **** p < 0.0001 (Mann-Whitney test).

**Extended Data Fig. 6:**
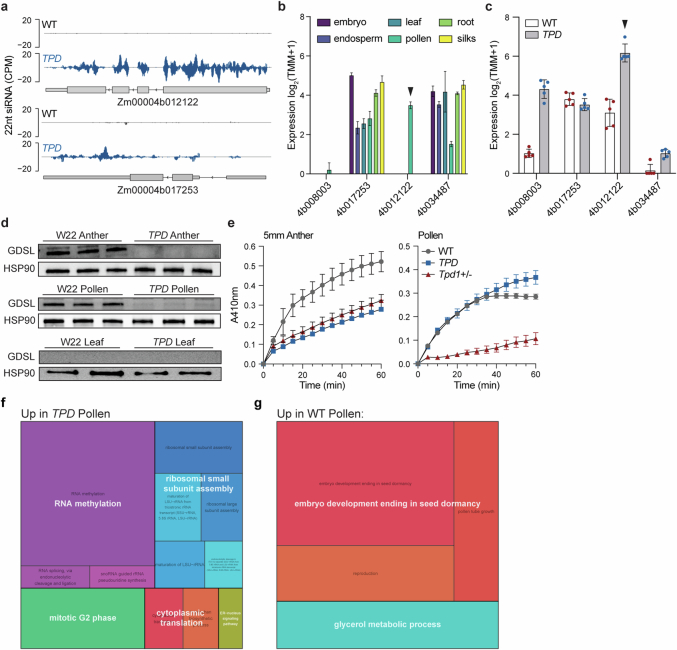
*TPD*-dependent silencing of a GDSL lipase disrupts lipid metabolism. **a**, Browser shots showing ectopic accumulation of 22-nt siRNAs at protein-coding genes in *TPD* pollen. Scale in counts per million (CPM). **b**, RNA-seq tissue expression of 22-nt siRNA targets specific to *TPD* pollen, data from ref. ^125^. Bars show mean ± SD. n = 3 replicates per tissue. **c**, RNA-seq expression of 22-nt siRNA targets in WT and *TPD* pollen. Bars show mean ± SD. n = 5 replicates per condition. **d**, Western blot comparing TDR1 protein levels in WT and *TPD* pollen, anthers, and leaf. Protein levels were normalized using Heat Shock Protein 90 (HSP90). **e**, p-nitrophenyl butyrate esterase activity assay in 5 mm anthers and pollen from WT, *TPD*, and *Tpd1* + */−* plants. **f, g**, GO term biological processes up-regulated in **f**, *TPD* and **g**, WT pollen (FDR ≤ 0.001). Upregulated genes in *TPD* pollen were associated with RNA metabolism, ribosome assembly, and cytoplasmic translation as well as G2 mitotic arrest. This could reflect translational repression via 22-nt siRNAs. Interestingly, a subset of genes associated with endoplasmic reticulum (ER)-nucleus signalling was also up-regulated^126^, while genes associated with glycerol metabolism, the primary backbone for TAG synthesis, were downregulated. In pollen, the accumulation of TAGs in lipid droplets (LDs) is critical for proper membrane expansion and pollen tube growth^127^.

In *TPD* pollen, we observed the accumulation of 158 ectopic 22-nt siRNA clusters across the genome (log_2_ fold change ≥ 2, FDR ≤ 0.01; Supplementary Table 5), and a general upregulation of genes associated with 22-nt siRNA biogenesis and function (Extended Data Fig. 5a). Nearly 60% of all ectopic 22-nt siRNAs in *TPD* pollen targeted transposable elements of the *P Instability Factor* (PIF)/Harbinger superfamily (Extended Data Fig. 5b), whose expression was *TPD* specific (Extended Data Fig. 5c–e). This superfamily is also activated following intraspecific hybridization and anther culture in rice^48^. However, a subset of protein-coding genes was also targeted in *TPD* pollen specifically (Extended Data Fig. 5b). Given that a reduction in 22-nt siRNAs suppressed the *TPD* phenotype, we hypothesized that inappropriate silencing of these genes might disrupt male reproductive development. In total, we identified four genes that gained ectopic 22-nt siRNAs in *TPD* pollen, approximately 62% of which came from a single gene (Zm00004b012122) that is also located on chromosome 5S (Extended Data Fig. 6a). Relative to other targets, this gene exhibited highly specific expression in pollen (Extended Data Fig. 6b,c). Zm00004b012122 encodes a GDSL triacylglycerol lipase/esterase, defined by a core catalytic sequence motif (GDSxxDxG), with roles in lipid metabolism, host immunity and reproductive development^49^. In maize, both *male sterile 30* (*ms30*) and *irregular pollen exine 1* (*ipe1*) mutants disrupt genes encoding a GDSL lipase and are completely male sterile^50,51^. Similar functions have been reported in rice^52^ and *Arabidopsis*^53^.

**Extended Data Fig. 7:**
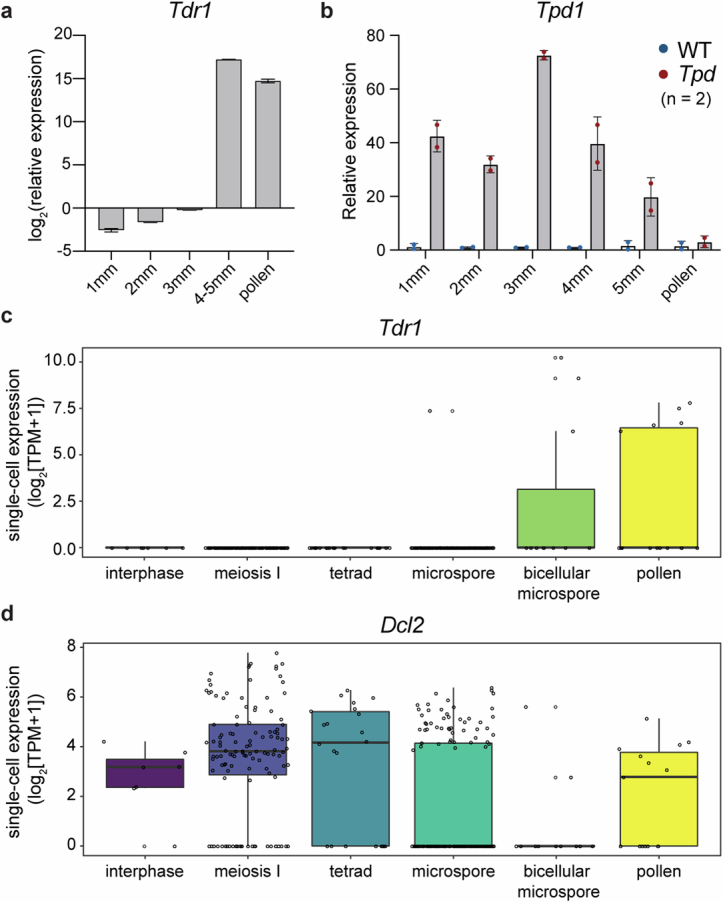
*Tpd1* and *Dcl2* are expressed pre-meiotically, whereas *Tdr1* is expressed in microspore and pollen. **a**, RT-qPCR of the *Tdr1* transcript throughout anther development and in mature pollen. Bars show mean ± SD. n = 2 replicates per condition. **b**, RT-qPCR of the *Tpd1* transcript during anther development and in mature pollen in WT and *Tpd*. Bars show mean ± SD. n = 2 replicates per condition. **c, d**, Single-cell expression at different stages of meiosis of **c**, *Tdr1* and **d**, *Dcl2*, using single cell RNAseq data^59^. Early and late expression of *Dcl2* coincides with *Tpd1* and *Tdr1*, respectively.

DCL2-dependent 22-nt siRNAs engage primarily in translational repression of their targets^54^, and consistently all four target genes had similar or higher levels of mRNA in *TPD* pollen (Extended Data Fig. 6c). We raised antiserum to the GDSL lipase for immunoblotting, choosing a surface-exposed peptide located between putative pro-peptide-processing sites reflecting endoplasmic reticulum localization^51^. The GDSL lipase protein accumulated strongly in both 5-mm anthers and mature pollen from wild-type plants, but was absent from leaf and from *TPD* anthers and pollen, supporting the conclusion that 22-nt siRNAs mediate translational repression (Extended Data Fig. 6d). Furthermore, whole-protein extracts from *TPD* anthers had reduced esterase activity, which was ameliorated in pollen containing *Tpd2* but not in pollen with *Tpd1* alone (Extended Data Fig. 6e). Gene ontology analysis of genes upregulated in wild-type and *TPD* pollen strongly supported translational suppression of the GDSL lipase as the primary cause of developmental arrest and abortion of pollen in *TPD* plants (Extended Data Fig. 6f,g). Finally, mRNA expression began post-meiotically at the 3-mm (tetrad) stage, peaking in 5-mm anthers and mature pollen (Extended Data Fig. 7a). This expression pattern was conspicuously similar to the developmental window in which *TPD* pollen abortion begins (Fig. 1f), suggesting that this gene might act as a ‘responder’ to *Tpd1-*driven distortion. On the basis of all these observations, we defined Zm00004b012122 as the primary candidate for targeting by *Tpd1* toxin activity, renaming it *Teosinte drive responder 1 (Tdr1)*.

## Hairpin siRNAs trigger pollen abortion

**Fig. 4:**
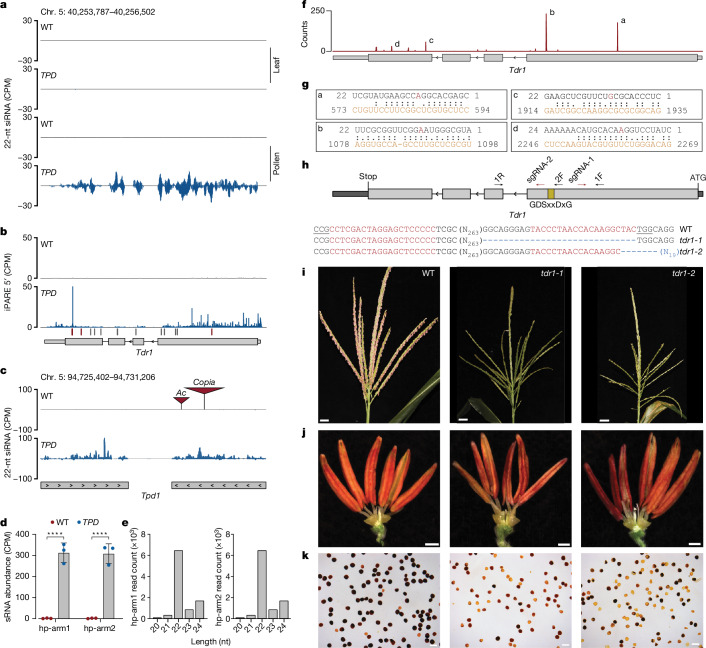
22-nt siRNAs from a *mexicana*-derived hairpin (*Tpd1*) target *Tdr1*, an essential pollen gene. **a**, 22-nt siRNA levels at the *Tdr1* locus in leaf and pollen tissue from WT and *TPD* genotypes. Ectopic 22-nt siRNAs accumulate in *TPD* pollen specifically. CPM, counts per million. **b**, iPARE-seq depicting the accumulation of 3′-OH cleavage products at the *Tdr1* locus. Tick marks indicate predicted target sites for hp-siRNAs derived from the *Tpd1* hairpin. Sites with (red) and without (grey) iPARE read support are shown. **c**, 22-nt hp-siRNA accumulation at the *Tpd1* hairpin. The hairpin locus is disrupted by transposable element insertions in the W22 genome. Data shown are normalized CPM (panels **a**–**c**). **d**, 22-nt hp-siRNA abundance at the *Tpd1* hairpin locus in WT and *TPD* pollen. *n* = 3 replicates per condition. ****P < 0.0001 (Mann–Whitney test). **e**, Average size distribution of reads mapping to the *Tpd1* hairpin. **f**, Small RNA target site prediction at the *Tdr1* locus using psRNATarget. Counts indicate unique hp-siRNAs from *Tpd1* that target each cleavage site. **g**, Homology between the guide strand (black) and the target strand (orange) is shown for the four most abundant hp-siRNAs. The tenth (red) and eleventh nucleotides in the guide strand flank the site of AGO-mediated cleavage. *Tpd1*-hp-siRNAb is predicted to suppress translation. **h**, CRISPR–Cas9 targeting of the *Tdr1* locus. Edits corresponding to *tdr1-1* and *tdr1-2* (blue) are shown. 1F, 1R, PCR primers; sgRNA, single guide RNA. **i**, Developmentally synchronized tassels from WT and *tdr1*-mutant T0 plants. *tdr1* mutants exhibit severely delayed anthesis. Scale bars, 3 cm. **j**, Mature 5-mm anthers from WT and *tdr1*-mutant T0 plants. Scale bars, 1 mm. **k**, I_2_KI viability staining of pollen from WT and *tdr1*-mutant T0 plants. Scale bars, 0.1 mm.

As ectopic silencing at protein-coding genes only occurred in the presence of the *Tpd1* haplotype, we reasoned that the distorter must generate small RNAs capable of triggering silencing in *trans*. In plants, microRNAs, secondary siRNAs and hp-siRNAs all have this capacity^55^. Processed small RNA duplexes are loaded into ARGONAUTE (AGO) proteins, passenger strands are released and RNase H-like slicing activity is targeted by guide strand homology, as is translational repression^56^. Silencing can be amplified via the coordinated action of RDR6 and SGS3 (ref. ^42^). RNase H-mediated slicing results in an exposed 5′-phosphate that allows for ligation of 3′ cleavage products. Using an improved degradome sequencing technique in *TPD* pollen, iPARE-seq (see Methods), we identified putative cleavage sites responsible for triggering silencing at the *Tdr1* locus (Fig. 4a,b). We simultaneously searched for non-coding RNA within the *Tpd1* haplotype that produced 22-nt sRNAs capable of triggering silencing. This approach yielded only one candidate: a large hpRNA similar to those identified previously in wild-type pollen (Fig. 4c). This hairpin was uninterrupted in the *mexicana*-derived *Tpd1* interval and produced high levels of *TPD*-specific 22-nt hp-siRNAs (Fig. 4d,e). In the W22 genome, we identified two large transposon insertions that interrupted this locus, which produced no small RNA, indicating that it was non-functional in maize, consistent with being responsible for *TPD* (Fig. 4c). By comparison with centromere placement in other maize inbreds^4^, the hairpin is on the short arm of chromosome 5, 5 Mb from the centromere.

Target site prediction uncovered four abundant hp-siRNA species predicted to target the *Tdr1* transcript in *trans* (Fig. 4f) resembling ‘proto-microRNA’^57^. Three of these began with 5′-C, indicating loading into Ago5, and had iPARE-seq support, indicating cleavage of *Tdr1* (Fig. 4g). However, the most abundant hp-siRNA, *Tpd1-*siRNAb, was 22 nt in length and began with 5′-A, indicating loading into Ago2 (Fig. 4g). *Tpd1-*siRNAb has an asymmetric bulge predicted to enhance silencing transitivity and systemic spread between cells^58^, and had only limited iPARE-seq support, indicating translational repression (Fig. 4b). To confirm that silencing of *Tdr1* was responsible for the *TPD* phenotype, we generated two independent frameshift alleles within the catalytic domain using CRISPR–Cas9 (Fig. 4h). Homozygotes for *tdr1-1* and *tdr1-2* had identical male sterile phenotypes, with extensive pollen abortion that phenocopied *Tpd1* (Fig. 4i–k).

**Extended Data Fig. 8:**
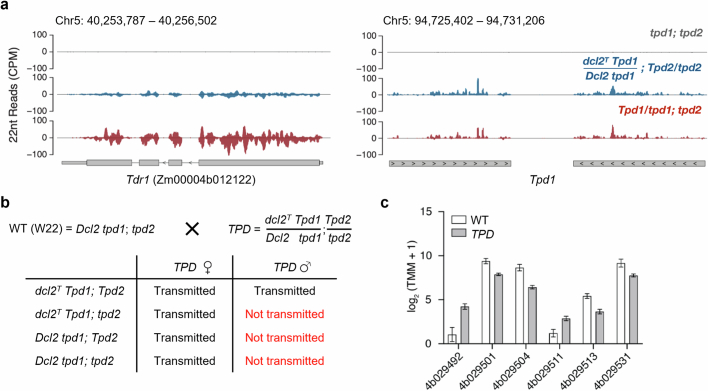
*Tpd2* suppresses 22nt secondary small RNAs. **a**, Browser shots showing ectopic accumulation of 22nt siRNAs at *Tdr1* (left) and *Tpd1* hairpin (right) in fertile (*tpd1; tpd2*, grey), drive (*Dcl2*^*T*^ *Tpd1/Dcl2 tpd1; Tpd2/tpd2*, blue) and sterile (*Dcl2 Tpd1/Dcl2 tpd1; tpd2*, red) pollen from maternal segregants. Scale in counts per million (CPM). *Tpd2* and *Dcl2*^*T*^ reduce small RNAs from *Tdr1* (left) but not from the *Tpd1* hairpin (right), consistent with a cell autonomous role in secondary small RNA biogenesis and silencing. **b**, Table summarizing genotypes transmitted when *TPD* is backcrossed to W22 as female (left column) or male (right column). Only the combination of *dcl2*^*T*^ *Tpd1* (linked) and *Tpd2* is transmitted through pollen. Recombinants between *dcl2*^*T*^ and *Tpd1* occur at the expected frequency but are not transmitted through pollen (Fig. 3b), presumably because of higher siRNA production during and after meiosis. **c**, *Rdm1* (Zm00004b029511) is one of six genes in the *Tpd2* interval expressed in pollen, and is overexpressed in *TPD* pollen.

Expression of the *Tpd1* hairpin was observed pre-meiotically in 1–3-mm anthers, as well as in microspores (4-mm anthers) where expression of *Tdr1* was first detected, but not in mature pollen (Extended Data Fig. 7b,c). According to published single-cell RNA sequencing data from developing maize pollen^59^, *Dcl2* is also expressed pre-meiotically, consistent with its role in generating 22-nt hp-siRNA from *Tpd1* (Extended Data Fig. 7d). *Dcl2* is not expressed in bicellular microspores, but is expressed in mature pollen consistent with an additional function in production of secondary small RNAs from *Tdr1* (Extended Data Fig. 7d). These results indicate a sequential order of events, in which expression of *Tpd1* pre-meiotically deposits small RNAs in microspores where they target *Tdr1*. Subsequent expression of *Dcl2* in mature pollen then promotes secondary small RNA production and translational suppression. Identification of *Tdr1* provided insight into the function of *Tpd2*. *Tpd1* hp-siRNAs were unaffected by *Tpd2*, which was instead required to suppress secondary small RNA biogenesis from *Tdr1*, along with the *mexicana* allele of *Dcl2*, namely, *dcl2*^*T*^ (Extended Data Fig. 8a). This indicates that *Tpd2* and *dcl2*^*T*^ have additive effects on suppressing secondary small RNAs, consistent with their role as partial antidotes (Extended Data Fig. 8b). Although the molecular identity of *Tpd2* remains unknown, the 1.5-Mb *Tpd2* interval contains six pollen-expressed genes in W22 (Extended Data Fig. 8c). One of these genes encodes the maize homologue of *Arabidopsis* RNA-DIRECTED DNA METHYLATION (RDM1), a critical component of the RNA-directed DNA methylation pathway^60^. This gene is significantly overexpressed in *TPD* pollen (Extended Data Fig. 8c), and it is possible that increased activity of RNA-directed DNA methylation might compete with the production of secondary small RNAs^61,62^, although further experimentation is required to support this idea.

## *TPD*, RNAi and the origin of modern maize

Population-level studies of maize traditional varieties identified an uninterrupted *mexicana*-derived haplotype surrounding the centromere of chromosome 5 (refs. ^32,63^) with high rates of linkage disequilibrium^63^. Consistent with reduced recombination, fine-mapping of *Tpd1* yielded very few informative recombinants (21 of 7,549) and none proximal to the hairpin (Extended Data Fig. 1c). Comparative analysis of the *TPD* and W22 genomes revealed three megabase-scale inversions, one of which corresponded to a 13-Mb event within the *Tpd1* haplotype and including *Bt1* on chromosome 5L (Fig. 2f,g). The presence of this inversion, along with its physical proximity to the centromere, explained our mapping data (Extended Data Fig. 1c) and strongly suggested that the *Tpd1* haplotype behaves as a single genetic unit.

The 13-Mb paracentric inversion in the *Tpd1* haplotype (W22 chromosome 5: 115,316,812–124,884,039) almost entirely encompasses ‘region D’ adjacent to centromere 5 (W22 chromosome 5: 118,213,716–126,309,970), which has undergone a dramatic domestication sweep in all maize inbreds relative to teosinte^4^. This region includes *Bt1*, which undergoes drive in the TPD system (Extended Data Fig. 1c). Our synthetic hybrids with maize inbred W22 retained approximately 13 intervals of the *mexicana* genome that persisted in serial backcrosses (Extended Data Fig. 2a,b). Four of these intervals are tightly linked to genes encoding AGO proteins, specifically *Ago1a*, *Ago1b*, *Ago2b* and *Ago5b*, all of which are expressed in the male germ line (Extended Data Fig. 2). According to 5′ nucleotide analysis, these AGO proteins are predicted to bind to *Tpd1*-hp-siRNAa–d (Ago2 and Ago5), as well as secondary *Tdr1* 22-nt siRNAs (Ago1), and it is conceivable that hypomorphic alleles could also act as partial antidotes in combination with *Tpd2*. In addition to intervals encoding *Dcl2*, *Rdm1* and *Rgd1/Sgs3*, this means that 7 of the 13 intervals are tightly linked to genes required for RNAi. These correlations suggest that there has been strong selection on all of these modifiers to ameliorate the toxic effects of *Tpd1*, resulting in apparent gene drive.

**Fig. 5:**
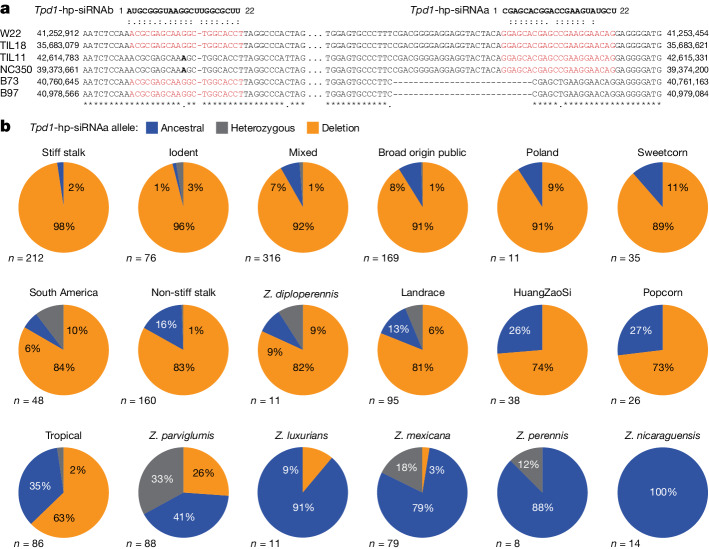
*Tpd1* hp-siRNA target site deletion in *tdr1* has spread to modern maize from teosinte. **a**, Sequence complement of the *Tpd1*-hp-siRNAa and *Tpd1*-hp-siRNAb target sites in *Tdr1*, indicating a 27-bp in-frame deletion found in modern maize, maize traditional varieties and in teosinte that removes the *Tpd1*-hp-siRNAa seed sequence, and a SNP on the eleventh nucleotide of *Tpd1*-hp-siRNAb that is predicted to reduce binding. **b**, Pie charts indicating the frequency of the deletion in 1,483 resequenced genomes from maize and teosinte, aligned with the B73 reference genome (GATK 3.0). The deletion allele (blue) arose in teosinte and quickly spread through maize traditional varieties in Central and South America, before fixation in modern stiff stalk, but not in tropical maize inbred lines. High frequencies of heterozygosity in *mexicana* and *parviglumis* are consistent with recent or ongoing pollen drive.

**Extended Data Fig. 9:**
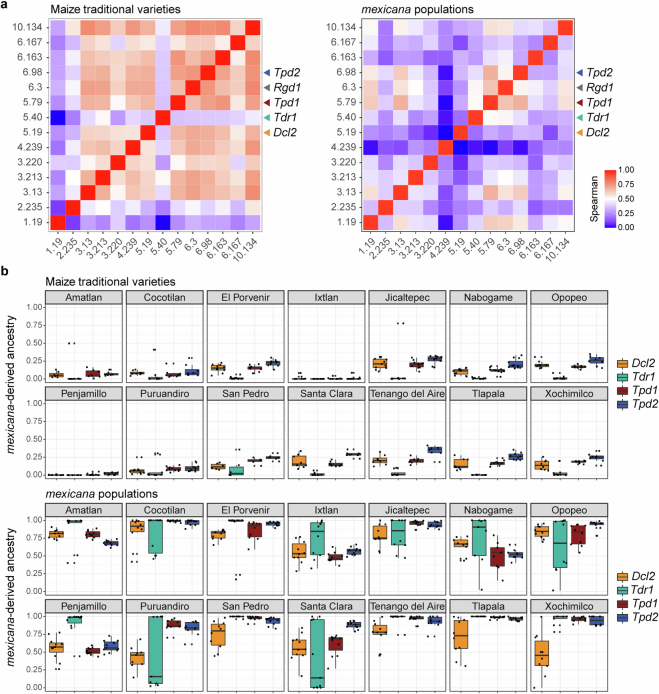
Signatures of *Teosinte Pollen Drive* in modern maize, maize traditional varieties and sympatric mexicana populations. **a**, Frequency of mexicana-derived alleles were calculated for 1 Mb intervals associated with *TPD* on chromosomes 1,2,3,4,5,6 and 10. Correlations are shown between population means from each of 14 maize traditional varieties (left) and sympatric *mexicana* populations (right). Intervals on chromosomes 5, 6 and 10 include *Dcl2* (5.19), *Tdr1* (5.40), *Tpd1* (5.79), *Rgd1/Sgs3* (6.3) and candidate genes *Ago1a* (6.3), *Tpd2/Rdm1* (6.98), *Ago1b* and *Ago2b* (10.134). Correlations were observed for most of the intervals in maize traditional varieties, except for *Tdr1* (green arrow), but only for intervals including *Tpd1*, *Rgd1* and *Tpd2* in *mexicana*. Spearman correlation coefficients are displayed as a heatmap. **b**, *mexicana*-derived ancestry in each of 14 maize traditional varieties (above) and sympatric *mexicana* populations (below) in *Dcl2*, *Tdr1, Tpd1* and *Tpd2* intervals. The *Tdr1* interval (green) is monomorphic in most of the maize traditional varieties, but shows extreme dimorphism in 7 out of 14 sympatric *mexicana* populations.

In traditional maize varieties, but not in sympatric *mexicana*, significant correlations were observed in *mexicana* ancestry between 11 of the 13 intervals (Extended Data Fig. 9a, Supplementary Tables 6 and 7 and Supplementary Discussion). By contrast, variation at *Tdr1* displays no such correlation with the co-inherited intervals in traditional maize varieties (Extended Data Fig. 9a). In fact, *Tdr1* is strongly monomorphic in traditional maize varieties, whereas in *mexicana*, *Tdr1* displays extreme polymorphism (Extended Data Fig. 9b). We considered the possibility that this locus has evolved to become immune to silencing in modern maize, a predicted outcome of selfish genetic systems^11^. A recent survey of maize and teosinte genome sequences^64^ has revealed that three of the four *Tpd1*-hp-siRNA target sites in *Tdr1* exhibit extensive polymorphism in maize and teosinte, including an in-frame deletion of the target site seed region for *Tpd1*-hp-siRNAa and a SNP at position 11 in target sites for *Tpd1-*hp-siRNAb, which are predicted to reduce or abolish cleavage and translational inhibition, respectively (Fig. 5a). *TPD* pollinations of the temperate inbred B73, which carries the deletion haplotype, resulted in 50% partially sterile (44 of 83) and fully fertile (35 of 83) offspring in advanced backcrosses, as well as rare fully sterile presumptive recombinants (4 of 83), consistent with these expectations. Surveys of the frequency of the deletion haplotype across *Zea* found it widespread, suggesting an origin before speciation of *Z. mays* from *Zea luxurians* and *Zea diploperennis* (Fig. 5b), whereas it is absent from *Zea* *nicaraguagensis* and *Tripsacum dactyloides*. The frequency of the deletion haplotype is relatively low in *mexicana* (12%) compared with *parviglumis* (46%), and increases in tropical maize, traditional maize varieties, popcorn and inbreds, where it is nearly fixed in several modern inbred groups (98%), suggesting a trajectory of spread to North and South America.

## Discussion

*TPD* is a toxin–antidote system that defies Mendelian inheritance and may have a history of selfish evolution, like other hybrid incompatibilities that cause gamete killing. Unlike teosinte crossing barriers *tcb-1*, *Ga-1* and *Ga-2* (ref. ^65^), which prevent fertilization, *TPD* resembles BDM incompatibility (also known as Dobzhansky–Muller incompatibility or DMI) in that it acts post-zygotically, resulting in sterile progeny. In canonical BDM, however, hybrid sterility is due to the unmasking of deleterious alleles, so that fertility eventually recovers in recurrent backcrosses to either parent. In *TPD*, backcrosses to maize result in pollen abortion no matter how many backcross generations are observed. This is because *TPD* is a special case of BDM that is consistent with meiotic drive. For gamete killers to spread via meiotic drive, they must compensate somehow for loss of fertility^66^. Loss of fertility may have posed a challenge for the spread of *TPD* in populations of teosinte. Therefore, establishing the evolutionary origin of *TPD* by meiotic drive will require additional population-level data and modelling, so that other explanations for gamete killing can be excluded^67^. In practice, maize–teosinte hybrids are extremely vigorous with numerous tassels, so that these wind-pollinated species may be less sensitive to reductions in male fertility. This is especially true during domestication, when early domesticates are typically less prolific than wild relatives, and at lower population size. In such circumstances, segregation distortion in hybrids could affect patterns of introgression between maize and teosinte.

**Extended Data Fig. 10:**
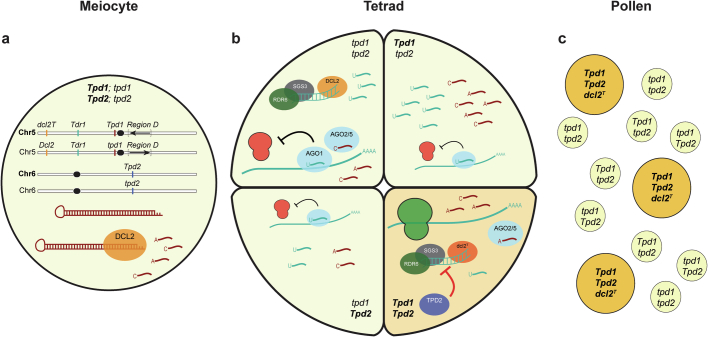
Mechanistic model of *Teosinte Pollen Drive.* **a**, The *TPD* system is defined by *mexicana* introgression intervals on chromosomes 5 and 6. *Tpd1* encodes a pre-meiotically expressed *mexicana*-specific hairpin that produces abundant 22nt hp-siRNAs. **b**, These hp-siRNAs trigger secondary siRNAs amplification by RDR6 and SGS3/RGD1 at the *Tdr1* gene when it starts being transcribed at the late tetrad stage, which in turn target *Tdr1* for translational repression (red ribosomes). In surviving microspores (dark yellow background) *Tpd2* and *dcl2*^*T*^ repress secondary siRNAs processing, restoring translation and fertility (green ribosome). **c**, Only pollen grains of the genotype *dcl2*^*T*^ *Tpd1*; *Tpd2* are viable, and all other competing gametes are eliminated. Other RNAi genes (*Sgs3*/*Rgd1*, *Ago1*, *Ago2*, *Ago5*) can act as partial suppressors by affecting levels of siRNAs.

**Extended Data Fig. 11:**
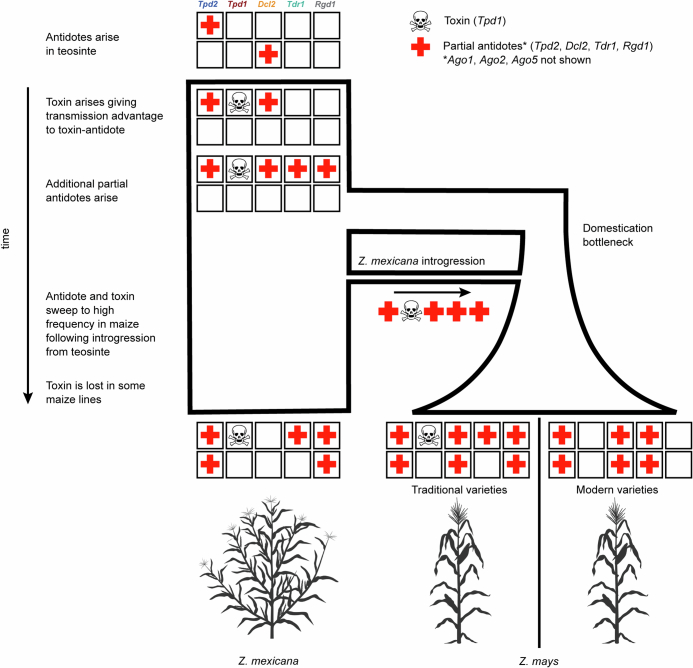
Evolutionary model of *Teosinte Pollen Drive.* After the antidotes arise in an ancestral teosinte population, the *Tpd1* toxin arises and gains a transmission advantage when linked to the antidote genes. In extant populations of *Z. mexicana* and *Z. mays*, some antidotes are fixed, while others are polymorphic or lost. The demographic model was based on ref. ^128^ and the conceptual framework of selfish evolution was adapted from ref. ^67^. Graphics were created using BioRender (https://biorender.com).

*Tpd1* encodes a long non-coding hpRNA that produces specific 22-nt hp-siRNAs in the male germline and kill pollen grains by targeting the genetically linked responder gene *Tdr1* (Extended Data Fig. 10a,b). This effect is countered by at least two gametophytic antidotes: a linked hypomorphic allele of *Dcl2* and the unlinked *Tpd2* locus on chromosome 6 (Extended Data Fig. 10c). The genetic architecture of this system, consisting of multiple linked and unlinked loci, deviates from previously established toxin–antidote systems. In rice, for instance, the *qHMS7* quantitative trait locus is a selfish genetic element composed of two tightly linked open reading frames^34^. Similarly, the *wtf4* driver in *S. pombe* features two alternatively spliced transcripts derived from the same locus^24^. By contrast, the *Tpd1* haplotype results from tight pseudolinkage between *Tpd1*, *Tdr1* and *dcl2*^*T*^ on chromosome 5, but only when transmitted through the male (Extended Data Fig. 8b). Although recombinants occur in single-pollen grains, they are not transmitted to the next generation (Fig. 1), and maternal recombinants between *dcl2*^*T*^ and *Tpd1* are completely male sterile (Fig. 2). These recombinants produce far more secondary 22-nt small RNAs at *Tdr1* (Extended Data Fig. 8a), providing an explanation for the failure to transmit recombinants through pollen. *Tpd2* is unlinked but acts cell autonomously, so that independent assortment of *Tpd1* and *Tpd2* occurs in female gametes, but never in male, implying that gametophytic suppression of pollen killing requires co-segregation of *Tpd2* with *Tpd1*. Although unlinked suppressors are relatively rare, a similar system has been reported in fission yeast^68^. In both cases, the selective suppression of drive can be interpreted as selfish behaviour on the part of the antidote. Ultimately, cycles of suppression and counter-suppression can be expected to result in complex, polygenic drivers that exist in a continuum of cryptic states (Extended Data Fig. 11), and the conspicuous maintenance of *mexicana* introgression intervals containing RNAi factors supports this idea (Extended Data Figs. 2a and 11).

Genome scans of sympatric maize and *mexicana* have identified multiple regions of introgression associated with adaptive variation, some of which overlap with the genomic interval corresponding to the *Tpd1* haplotype^32^ and other intervals undergoing distortion^69^, and we found that intervals associated with drive in pollen are significantly correlated with each other in maize traditional varieties, but not in sympatric *mexicana* populations (Extended Data Fig. 9). We postulated that the most powerful suppressor of all would be an ‘immune’ target gene, in which hp-siRNA target sites in *Tdr1* had been mutated. Such in-frame immune haplotypes were found in wild taxa in *Zea* and have been progressively fixed from tropical to temperate stiff-stalk maize inbreds (Fig. 5), suggesting that *TPD* may be an ancient system that has influenced admixture throughout the history of the genus, reaching fixation in modern maize. *TPD* complements the hypothesized role of *Ab10*, a chromosomal driver of female meiosis that simulations suggest may have been responsible for the redistribution of heterochromatic knobs in maize, *parviglumis* and *mexicana*^15,70^, potentially along with thousands of linked genes^16^.

Our results suggest that DCL2-dependent 22-nt small RNAs stemming from long hpRNAs function as selfish genetic elements in pollen. In *Arabidopsis*, 22-nt siRNA biogenesis is carefully regulated due to ectopic silencing of host genes^37,40,42,54^, and 21–22-nt siRNAs from pollen mediate triploid seed abortion^71,72^ and can block self-fertilization^73^. In *Drosophila melanogaster*^74,75^, silencing of protein-coding genes by recently evolved hairpins is important for male reproductive development^75^, whereas in *Drosophila* *simulans*, the Winters sex-ratio distortion system is actually suppressed by two hpRNAs, *Not much yang* (*Nmy*) and *Too much yin* (*Tmy*), which act as antidotes and are essential for male fertility and sex balance^76,77^. In mammals, endo-siRNAs in the oocyte are generated from hairpin and antisense precursors by an oocyte-specific Dicer isoform (*Dcr-O*) and have an essential function in global translational suppression^78–80^. The remarkable parallels between all of these systems, and between Dcr-O and *dcl2*^*T*^, which both have potential defects in the helicase domain, invites speculation that selection for selfish behaviour is an efficient means by which germline small RNAs can propagate within a population. Such propagation provides a plausible origin for ‘self’-targeting small RNAs in the germlines of plants and animals.

## Methods

### Plant material and growth conditions

The *TPD* lineage traces to teosinte *mexicana* collected near Copándaro, Michoacán, Mexico in December 1993. Gamete a, plant 4 of collection 107 was used in an initial outcross to the Midwestern US dent inbred W22 and subsequently backcrossed. *Tpd1;Tpd2* (BC_8_S_3_) homozygous lines were used for whole-genome sequencing and de novo genome assembly. All additional experiments were performed using *Tpd1/tpd1; Tpd2/tpd2* (BC_11_–BC_13_) plants or populations derived from maternal segregation of these lines. The *lbl1-rgd1* and *dcl2-mu1* alleles were backcrossed to W22 four or more times. *dcl2-mu1* was isolated from the Uniform-Mu line UFMu-12288. All genetic experiments used segregating wild-type progeny as experimental controls. Plants were grown under greenhouse and field conditions.

### Phenotyping and microscopy

All pollen phenotyping was performed using mature 5-mm anthers before anthesis. Individual anthers were suspended in PBS and dissected using forceps and an insulin syringe. Starch viability staining was performed using Lugol solution (L6146-1L, Sigma). Measurements for days to anthesis were taken for three replicate crosses (*Tpd1/tpd1;Tpd2/tpd2* × W22) with staggered planting dates in three different field positions. The leaf collar method^81^ was combined with routine manual palpation of the topmost internode to track reproductive stages. Meiotic anthers were dissected, fixed in 4% paraformaldehyde plus MBA buffer^82^, and stained with DAPI for visualization. For tetrad viability assays, anthers from the upper floret of an individual spikelet were dissected and stored in MBA. One anther was used for staging and the others were dissected to release the tetrads. FDA viability staining was performed as previously described^83^. To control for artefacts associated with sample handling, only intact tetrads (four physically attached spores) were considered.

### Genotyping and marker design

For routine genotyping, tissue discs were collected with a leaf punch and stored in 96-well plates. To extract genomic DNA, 20 μl of extraction solution (0.1 M NaOH) was added to each well and samples were heated to 95 °C for 10 min and then placed immediately on ice. To neutralize this solution, 90 μl of dilution solution (10 mM Tris + 1 mM EDTA, pH to 1.5 with HCl) was added. PCRs, using 1–2 μl of this solution as template, were performed using GoTaq G2 Green Master Mix (M7822, Promega). Secondary validation of genotyping reactions was performed as needed using the Quick-DNA Plant/Seed Miniprep kit (D6020, Zymo Research). Bulk Illumina and Nanopore data from *Tpd1;Tpd2* seedlings was used for co-dominant molecular marker design (Supplementary Table 8). When possible, markers based on simple sequence length polymorphisms were prioritized, but a number of restriction fragment length polymorphisms were also designed. W22, *Tpd1/tpd1;Tpd2/tpd2* and *Tpd1;Tpd2* genomic DNA was used to validate marker segregation before use. The *dcl2-mu1* insertion was amplified by combining gene-specific forward and reverse primers with a degenerate terminal inverted repeat primer cocktail. The insertion was subsequently validated by Sanger sequencing.

### High-molecular-weight genomic DNA extraction

High-molecular-weight (HMW) genomic DNA was used as input for all Nanopore and bulk Illumina sequencing experiments. For extraction, bulked seedlings were dark treated for 1 week before tissue collection. Four grams of frozen tissue was ground under liquid N_2_ and pre-washed twice with 1.0 M sorbital. The tissue was then transferred to 20 ml pre-warmed lysis buffer (100 mM Tris-HCl (pH 9.0), 2% w/v CTAB, 1.4 M NaCl, 20 mM EDTA, 2% PVP-10, 1% 2-mercaptoethanol, 0.1% sarkosyl and 100 μg ml^−1^ proteinase K), mixed gently and incubated for 1 h at 65 °C. Organic extraction in phase-lock tubes was performed using 1 vol phenol:chloroform:isoamyl alcohol (25:24:1) followed by 1 vol chloroform:isoamyl alcohol. DNA was precipitated by adding 0.1 vol 3 M NaOAc (pH 5.2) followed by 0.7 vol isopropanol. HMW DNA was hooked out with a pasteur pipette and washed with 70% EtOH, air dried for 2 min and resuspended in 200 μl Tris-HCl (pH 8.5; EB). The solution was treated with 2 μl 20 mg ml^−1^ RNase A at 37 °C for 20 min followed by 2 µl 50 mg ml^−1^ proteinase K at 50 °C for 30 min. 194 μl EB, 100 µl NaCl and 2 μl 0.5 M EDTA were added, and organic extractions were performed as before. DNA was precipitated with 1.7 vol EtOH, hooked out of solution with a pasteur pipette, washed with 70% EtOH and resuspended in 50 μl EB.

### Nanopore and Hi-C sequencing, *TPD* genome assembly and annotation

HMW DNA from *Tpd1;Tpd2* BC_8_S_3_ was gently sheared by passage through a P1000 pipette 20 times before library preparation with the Oxford Nanopore Technologies Ligation Sequencing gDNA (SQK-LSK109) protocol with the following modifications: (1) DNA repair, end-prep and ligation incubation times were extended to 20 min each; (2) 0.8× vol of a custom SPRI bead solution was used for reaction cleanups^84,85^; and (3) bead elutions were carried out at 50 °C for 5 min. Libraries were sequenced on the MinION device with R9.4.1 flow cells. Offline base calling of Oxford Nanopore Technologies reads was performed with Guppy 5.0.7 and the R9.4.1 450-bp super accuracy model. Reads longer than 1 kb were assembled into contigs using Flye 2.9-b1768 (ref. ^86^) with options ‘--extra-params max_bubble_length=2000000 -m 20000 -t 48 --nano-raw’. The same long reads were aligned to the Flye contigs (filtered to keep only the longest alternatives) using minimap2 2.22-r1101 (ref. ^87^), and these alignments were passed to the PEPPER-Margin-DeepVariant 0.4 pipeline^88^ to polish the initial consensus. To correct remaining single-nucleotide variants and small indels, two Illumina PCR-free genomic DNA PE150 libraries were mapped to the long read polished consensus with bwa-mem2 2.2.1 (ref. ^89^) for further polishing with NextPolish 1.3.1 (ref. ^90^) followed by Hapo-G 1.2 (ref. ^91^), both with default options. Two biological replicate samples of BC_8_S_3_ leaf tissue were used to prepare Dovetail Omni-C Kit libraries following the manufacturer’s protocol, and sequenced as a PE150 run on a NextSeq500. These Hi-C reads were mapped to the polished contigs with the Juicer pipeline release 1.6 UGER scripts with options ‘enzyme=none’^92^. The resulting ‘merged_nodups.txt’ alignments were passed to the 3D DNA pipeline to iteratively order and orient the input contigs and correct misjoins^93^. This initial automatic scaffolding resulted in 11 superscaffolds longer than 10 Mb. Correcting a single centromeric break during manual review with JBAT^94^ resulted in the expected 10 pseudomolecules. One 6-Mb contig was identified as bacterial with no contacts and was discarded. The remaining unscaffolded contigs were of organelle origin (*n* = 9, 625 kb) or aligned to the pseudomolecules (*n* = 116, 12 Mb). Coding gene predictions from the NRGene 2.0 W22 (ref. ^95^) were projected onto the *TPD* genome assembly using Liftoff 1.6.2 (ref. ^96^) with options ‘-polish -copies -chroms <chrom_map>’. An average Phred quality value (QV) score for the assembly was estimated from a 20-mer database of the Illumina reads using merqury 1.4.1 (ref. ^97^) with default options. Assembly completeness was also assessed with BUSCO 5.5.0 (ref. ^98^) with options ‘-m genome --miniprot’. See Supplementary Table 3 for assembly metrics.

### RNA extraction

Tissue was collected, snap frozen in liquid nitrogen and stored at −80 °C. Samples were ground into a fine powder using a mortar and pestle on liquid nitrogen. Of pre-extraction buffer (100 mM Tris-HCl (pH 8.0), 150 mM LiCl, 50 mM EDTA (pH 8.0), 1.5% v/v SDS and 1.5% 2-mercaptoethanol), 800 µl was added and mixed by vortexing. Of acid phenol:chloroform (pH 4.7–5.0), 500 µl was added and samples were mixed then spun down at 13,000*g* for 15 min at 4 °C. The aqueous layer was extracted, and 1 ml TRIzol per 200 mg input tissue was added. Samples were mixed by vortex and incubated at room temperature for 10 min. Chloroform (200 µl) per 1 ml TRIzol was added and samples were mixed by vortexing and then incubated at room temperature for 2 min. Samples were then spun down at 13,000*g* for 15 min at 4 °C. The aqueous phase was extracted and cleaned up using the Zymo RNA Clean and Concentrator-5 kit (R1013, Zymo Research). Only samples with RNA integrity scores of 9 or more were used for quantitative PCR (qPCR) and sequencing.

### Reverse transcription and RT–qPCR

For reverse transcription, 1 µg of total RNA was treated with ezDNase (11766051, Thermo Fisher) according to the manufacturer’s instructions. Reverse transcription was performed with SuperScript IV VILO Master Mix (11756050, Thermo Fisher). Following reverse transcription, complementary DNA (cDNA) was diluted 1:20 in dH_2_0 to be used as template in qPCR with reverse transcription (RT–qPCR).

All RT–qPCR experiments were performed on an Applied Biosystems QuantStudio 6 system in 96-well plate format using PowerUp SYBR Green Master Mix (A25741, Thermo Fisher). Before use in experiments, primer efficiency was tested for each primer set using a standard curve generated from serial dilutions of cDNA template. Only primer sets with efficiencies between 90% and 110% were used (Supplementary Table 9). For experiments, three or more biological replicates (independent cDNA samples from discrete plants) were assayed per genotype, and two or more technical replicates were set up for each reaction condition. Raw Ct (cycle threshold) from technical replicates were averaged, and ^∆^Ct (mean Ct^exp^ – mean Ct^ref^) was calculated using *Elfa9* as a housekeeping reference. ^∆∆^Ct values (^∆^Ct^cond1^ – ^∆^Ct^cond2^) were calculated between genotypes and converted to fold change (2^(^^–^^∆∆Ct)^).

### Whole-genome sequencing and SNP calling

For HMW DNA from separately maintained *Tpd1;Tpd2* lineages (BC_8_S_3_ and BC_5_S_2_) and from bulk segregation analysis maternal pools, extractions were as detailed above. Libraries were prepared using the Illumina TruSeq DNA PCR-Free kit (20015962, Illumina) with 2 μg of DNA input. Samples were sequenced on a NextSeq500 platform using 2 × 150-bp high-output run. Adapter trimming was performed with Cutadapt (v3.1)^99^. Paired-end reads were aligned to the W22 reference genome^95^ with BWA-MEM (v0.7.17)^100^. Alignments were filtered by mapping quality (mapQ ≥ 30), and PCR duplicates were removed using SAMtools (v1.10)^101^. SNP calling was performed using Freebayes (v1.3.2)^102^. Putative SNP calls were filtered by quality, depth and allele frequency (allele frequency = 1) to obtain a high-confidence *mexicana* marker set that was subsequently validated against the *TPD* assembly. For bulk segregation analysis^103^, SNP calls were filtered against the gold-standard *TPD* marker set. Reference and alternate allele frequencies at each marker were calculated and the average signal was consolidated into 100-kb bins. The ∆SNP index was then calculated for each bin in a sliding window.

### Single-pollen grain sequencing

Pollen grains from *Tpd1/tpd1;Tpd2/tpd2* plants were suspended in ice-cold PBS on a microscope slide under a dissecting scope. Individual plump, viable pollen grains were deposited into the 0.2-ml wells of a 96-well plate using a p20 pipette. Lysis and whole-genome amplification were performed using the REPLI-g single-cell kit (150345, Qiagen) with the following modifications: one-fourth of the specified volume of amplification mix was deposited in each well and isothermal amplification was limited to 5 h. All steps before amplification were performed in a UV-decontaminated PCR hood. Whole-genome analysis products were cleaned up using a Genomic DNA Clean & Concentrator kit (D4067, Zymo Research), and yields were quantified using with the QuantiFluor dsDNA system (E2670, Promega) in a 96-well microplate format.

Libraries were prepared using the TruSeq Nano DNA High Throughput kit (20015965, Illumina) with 200 ng input. Samples were sequenced on a NextSeq500 platform using 2 × 101-bp high-output runs. Quality control, adapter trimming, alignment and SNP calling were performed as above. BCFtools 1.14 (ref. ^104^) was used to derive genotype calls from single-pollen grains at the predefined marker positions and then passed to GLIMPSE 1.1.1 (ref. ^105^) for imputation. All calls at validated marker sites were extracted and encoded in a sparse matrix format (rows = markers, columns = samples) and encoded (1 = alt allele, −1 = ref allele, 0 = missing). To assess *mexicana* introgression in individual pollen grains, mean SNP signal was calculated in 100-kb bins across the genome. A sliding window (1-Mb window, 200-kb step) was applied to smooth the data and identify regions with *mexicana* SNP density. To identify genomic intervals overrepresented in surviving *TPD* pollen grains, aggregate allele frequency was calculated across all pollen grains at each marker site.

### RNA sequencing and analysis

Five biological replicates were prepared for each biological condition (*Tpd1/tpd1;Tpd2/tpd2* and *tpd1;tpd2* siblings). Of total RNA, 5 µg was ribosome depleted using the RiboMinus Plant Kit (A1083808, Thermo Fisher), and libraries were prepared using the NEXTFLEX Rapid Directional RNA-seq kit (NOVA-5138-08, PerkinElmer). The size distribution of completed libraries was assessed using an Agilent Bioanalyzer, and quantification was performed using a KAPA Library Quantification kit (KK4824, Roche). Libraries were sequenced on a NextSeq500 platform using a 2 × 150-bp high-output run. Trimmed reads were aligned to the W22 reference with STAR in two-pass alignment mode^106^. Read counts were assigned to annotated features using featureCounts^107^. For transposable element expression, multi-mapping reads were assigned fractional counts based on the number of identical alignments. Differential expression analysis was performed using edgeR^108^. To avoid false positives, a stringent cut-off (log_2_ fold change ≥ 2, FDR ≤ 0.001) was used to call differentially expressed genes. Gene ontology analysis (Fisher’s exact test, *P* < 0.01) was performed using topGO^109^, and the results were visualized using rrvgo^110^. For data visualization, alignment files were converted to a strand-specific bigwig format using deepTools^111^.

### Small RNA sequencing and analysis

For comparisons between *Tpd1/tpd1;Tpd2/tpd2* and *tpd1;tpd2* pollen, three biological replicates were used. Two biological replicates were used for *dcl2*^*T−/−*^ and *dcl2-mu1*^*−/−*^ pollen samples. Libraries were constructed with the NEXTFLEX Small RNA-Seq V3 kit (NOVA-5132-06, PerkinElmer) using 2 μg of total RNA input per library and the gel-free size selection protocol. The size distribution of completed libraries was assessed using an Agilent Bioanalyzer, and quantification was performed using a KAPA Library Quantification kit (KK4824, Roche). Libraries were sequenced on a NextSeq500 platform using a 1 × 76-bp run. Adapters were trimmed using cutadapt^99^, and the 4-bp unique molecular identifier sequences on either side of each read were removed.

Reads were filtered using pre-alignment to a maize structural RNA consensus database using bowtie2 (ref. ^112^). Alignment and de novo identification of small RNA loci were performed with ShortStack^113^, using a minimum CPM cut-off of 5, and only clusters with clear size bias (21, 22 or 24 nt) were retained in downstream analysis. Differential sRNA accumulation was performed with edgeR^108^ (log_2_ fold change ≥ 2, FDR ≤ 0.01). The accumulation of size and strand-biased hp-siRNAs was used to identify hairpin loci throughout the genome. For each locus, the underlying primary sequence was tested for reverse complementarity, and RNA secondary structure prediction was performed using RNAfold^114^. Non-hp-siRNA targets were only retained if they showed negligible strand bias (that is, evidence of a double-stranded RNA template for processing by a Dicer-like enzyme).

### iPARE-seq and analysis

iPARE-seq is an improvement on degradome sequencing by PARE-seq^115^. For iPARE-seq libraries, 40 μg of total RNA was poly(A) selected using a Dynabeads mRNA Purification Kit (61006, Thermo Fisher). Of poly(A) RNA, 1 µg was ligated to the 5′ PARE adapter (100 pmol) in 10% DMSO, 1 mM ATP, 1X T4 RNA ligase 1 buffer (B0216L, New England Biolabs), 25% PEG8000 with 1 μl (40U) of RNaseOUT (10777019, Thermo Fisher) and 1 μl T4 RNA ligase 1 (M0204S, New England Biolabs) in a reaction volume of 100 μl. Ligation reactions were performed for 2 h at 25 °C followed by overnight incubation at 16 °C. Samples were then purified using RNA Clean XP beads (A63987, Beckman Coulter) and eluted in 18 μl dH_2_0. Chemical fragmentation of ligated RNA to 200 nt or fewer was performed using the Magnesium RNA fragmentation kit (E6150S, New England Biolabs). Of RNA fragmentation buffer, 2 µl was added and samples were incubated at 94 °C for 5 min followed by a transfer to ice and the addition of 2 μl of RNA Stop solution. Samples were purified using the RNA Clean & Concentrator-5 kit (R1013, Zymo Research) and eluted in 11 μl H_2_0. Reverse transcription was performed as follows: 10 μl of RNA, 1 μl of 10 mM dNTP and 2 μl of random primer mix (S1330S New England Biolabs) were mixed and incubated for 10 min at 23 °C, and then put on ice for 1 min. The following was then added: 4 μl of 5X SuperScript IV buffer, 1 μl of 100 mM DTT, 1 μl of RNaseOUT and 1 μl of Superscript IV (200U). The reaction was incubated for 10 min at 23 °C, followed by 10 min at 50 °C. Of Tris-EDTA, 80 µl was then added to this mixture.

Target indirect capture was performed with 100 μl Dynabeads MyOne Streptavidin T1 beads (65601, Thermo Fisher) as per the manufacturer's instructions. Of the reverse transcription reaction, 100 µl was used as input, and captured cDNA molecules were eluted in 50 μl. Second-strand synthesis was performed using 5U Klenow fragment (M0210S, New England Biolabs) with 100 µM dNTPs and 1 μM of iPARE adapter primer (5′-NNNNTCTAGAATGCATGGGCCCTCCAAG-3′) for 1 h at 37 °C and incubation at 75 °C for 20 min. Samples were purified with a 1:1 ratio of AMPure XP SPRI beads (A63880, Beckman Coulter) and resuspended in 51 μl EB. Of sample, 50 µl was used for library preparation with the NEB Ultra DNA library kit (E7370S, New England Biolabs). Barcoded samples were sequenced with a NextSeq500 2 × 150-bp high-output run. Use of the directional iPARE adapter allows for the retention of directionality even when using a non-directional DNA-seq kit. Cutadapt^99^ was used to search and recover the adapter sequence in both 5′ and 3′ orientation (forward in read1 or read2, respectively). Read1 adapter reads were trimmed for the 3′ adapter if present, and the 5′ iPARE adapter was subsequently removed. Potential polyA tails were also removed, and only reads of 20 nt or more were retained. Read2 adapter reads were processed in an identical manner. Filtered reads were mapped using Bowtie2 (ref. ^112^) and the 5′ position of each read (the cloned 5′-monophosphate corresponding to the position of AGO-mediated cleavage) was extracted using BEDtools^116^ with CPM normalization. Small RNA target prediction was performed using psRNATarget^117^.

### Protein extraction and western blotting

Fresh anthers or pollen were collected and snap frozen in liquid nitrogen. Samples were then ground to a fine powder in a mortar and pestle over liquid nitrogen and resuspended in freshly prepared extraction buffer (2 mM Tris-HCl (pH 7.4), 150 mM NaCl, 1 mM EDTA, 1% v/v NP-40, 5% v/v glycerol, 1 mM PMSF and 1 ml Roche protease inhibitor cocktail per 30 g input tissue) and vortexed thoroughly. Samples were then centrifuged at 14,000 rpm at 4 °C for 5 min to pellet cellular debris, and the aqueous fraction was transferred to another tube. This step was then repeated twice more. Protein extracts were quantified using the Pierce Detergent Compatible Bradford Assay Kit (23246, Thermo Fisher) on a Promega Glomax-Multi+ plate reader.

To assess the role of 22-nt siRNAs in translational repression, antiserum was raised to a peptide (SRKGAPPSSPPLSPPKLGA) from the Zm00004b012122 protein in collaboration with PhytoAB. Specificity was determined as follows: (1) blots using pollen protein extracts showed a single band at roughly the expected size, and (2) blots using leaf protein extracts showed no band in concordance with expected pollen/anther specificity. A rabbit polyclonal HSP90-2 antibody (AS11 1629, Agrisera), a constitutive isoform with high expression, was used as loading control in all western blot experiments. For comparisons of protein abundance between wild-type and *TPD* pollen/anthers, 2 µg of protein was denatured at 95 °C for 5 min in an appropriate volume of 2X Laemmli buffer (120 nM Tris-Cl (pH 6.8), 4% v/v SDS, 0.004% bromophenol blue, 20% v/v glycerol, 0.02% w/v bromophenol blue and 350 mM DTT). Samples were run on a 4–20% Mini-PROTEAN TGX Precast Gel (4561094, Bio-Rad) with a Precision Plus Protein Dual Xtra Prestained standard (1610377, Bio-Rad).

Transfer to a PVDF membrane was performed using a Bio-Rad Trans-Blot Turbo Transfer system. Membranes were blocked using 5% w/v powdered milk in 1X TBS-T (20 mM Tris, 150 mM NaCl and 0.1% Tween-20) for 1 h at room temperature. Subsequently, the membrane was cut and incubated with primary antibody (1:3,000 dilution in blocking solution) at 4 °C overnight with gentle agitation. Three 15-min membrane washes were performed with 1X TBS-T at room temperature. Membranes were then incubated with a 1:3,000 goat anti-rabbit IgG H&L (PHY6000, PhytoAB) secondary antibody for 1 h at room temperature. Following three more washes with 1X TBS-T, membranes were incubated for 5 min with ECL Prime detection reagent (RPN2236, Amersham) and visualized using a Bio-Rad ChemiDoc Touch Imaging System.

### Esterase enzymatic activity assay

Esterase activity assays were performed using the colorimetric substrate *p*-nitrophenyl butyrate (N9876, Sigma) at a final concentration of 1 mM in 0.5 M HEPES (pH 6.5). For assays using whole 5-mm anthers, 100 μg of total protein was used as input for each sample, whereas 50 μg was used for pollen. Individual samples were prepared in cuvettes at a volume of 1.5 ml. Upon addition of the total protein extract, samples were gently mixed, and an initial 410-nm absorbance reading was taken to serve as a per sample baseline. Samples were then incubated at 30 °C, and absorbance readings were taken every 5 min for a total of 12 timepoints. This experiment was replicated three times for each genotype. All absorbance readings were taken using a Thermo Scientific Genesys 20 spectrophotometer.

### Detection of selective sweeps in candidate regions associated with *TPD*

We investigated signals of selection in genomic regions associated with *TPD* using selscan (v1.2.0a)^118^ to calculate the genome-wide normalized absolute integrated haplotype score (iHS) statistics for individual SNPs and in 10-kb windows. iHS is suitable for identifying selection in a single population and relies on the presence of ongoing sweeps and a signal of selection from unusually long-range linkage disequilibrium. We also used VCFtools (v0.1.16)^119^ to calculate Weir and Cockerham’s *F*_ST_ in 10-kb windows to assess signals of selection based on changes in allele frequency between populations. Phased SNPs for modern temperate maize lines, teosinte and *T. dactyloides* were obtained from Grzybowski et al.^120^, and SNPs for 265 CIMMYT traditional varieties were obtained from Yang et al.^121^ and phased with Beagle (v5.4)^122^. A phased and imputed set of 42,387,706 genome-wide concatenated SNPs was used for the analysis of selection. The *T*. *dactyloide*s allele was set to be the ancestral allele. A consensus genetic map curated by Ed Coe was obtained from MaizeGDB^123^, and SNP positions were interpolated to genetic positions. Weighted *F*_ST_ was calculated for each unique population pair. For iHS, 10-kb windows were binned into 10 quantiles based on the number of SNPs they contained, and empirical *P* values for each window were calculated within each quantile. The statistic calculated was the number of extreme (top 5%) |iHS| scores per window. Empirical *P* values for iHS and *F*_ST_ were then calculated from the rank of each window based on the respective statistics. We adjusted these *P* values for multiple testing of different populations using the Bonferroni method. *TPD*-linked regions (*dcl2*, *rdm1*, *tdr1* and hairpin region) and their 1-kb upstream and downstream regions were intersected with the 10-kb windows using bedtools (v2.30)^116^ and assigned the lowest *P* value of all intersecting windows. To validate our selection scan, we also investigated windows intersecting with a set of four known domestication genes^124^.

### Reporting summary

Further information on research design is available in the Nature Portfolio Reporting Summary linked to this article.

## Online content

Any methods, additional references, Nature Portfolio reporting summaries, source data, extended data, supplementary information, acknowledgements, peer review information; details of author contributions and competing interests; and statements of data and code availability are available at 10.1038/s41586-024-07788-0.

## Supplementary information

Supplementary Information Supplementary Discussion, Supplementary Tables 1–3 and Supplementary Tables 6–9

Reporting Summary

Supplementary Tables

Supplementary Table 4 22nt clusters enriched in WT pollen vs WT leaf

Supplementary Table 5 22nt clusters enriched in *TPD* pollen vs WT pollen

## Data Availability

Sequencing datasets generated during the current study are available at the NCBI (Gene Expression Omnibus SuperSeries: GSE234925). Datasets used for genome assembly are available at the Sequence Read Archive (BioProject: PRJNA937229). This Whole-Genome Shotgun project has been deposited at DDBJ/ENA/GenBank under the accession JARBIH000000000. The version described in this paper is version JARBIH010000000. All materials are available on request.

## Code availability

All code is available on Github (https://github.com/martienssenlab/TPD-manuscript).
