# Supplementary material for: Teosinte Pollen Drive guides maize diversification and domestication by RNAi
Source: Nature. 2024 Aug 7;633(8029):380–8. doi: 10.1038/s41586-024-07788-0 (PMC11390486; doi:10.1038/s41586-024-07788-0)
Supplement: Supplementary file 1 — Supplementary Discussion, Supplementary Tables 1–3 and Supplementary Tables 6–9 [file 41586_2024_7788_MOESM1_ESM.pdf]

nature portfolio

https://doi.org/10.1038/s41586-024-07788-0

Supplementary information

Teosinte Pollen Drive guides maize diversification and domestication by RNAi

In the format provided by the authors and unedited

Supplementary Discussion

for *Teosinte Pollen Drive* guides maize diversification and domestication by RNAi.

The *Tpd1* interval includes a large inversion

Meiotic recombination has been proposed to be an evolutionary consequence of defense against drive, as the separation of individual drive components is an efficient way to purge selfish genetic elements[129–131] and rare recombinants in the *SD* and *t*-complexes in mouse and Drosophila exhibit either impaired SD or suicidal male sterility[21,132]. As such, many drive complexes evolve in regions of reduced recombination and select for further structural rearrangements, some of which can give rise to patterns of variation that influence morphological traits not associated with drive itself[11]. The *t*-complex, which contains genetic loci governing SD, embryonic lethality, and morphological phenotypes such as tail length is a clear example[132]. At the structural level, the *Tpd1* haplotype is consistent with this notion, encompassing both the core centromeric region and a 13Mb paracentric inversion on chromosome 5L. In *mexicana*, the *Tpd1* haplotype captures upwards of 243 protein-coding genes including at least 12 putative loss of function alleles, significant levels of nonsynonymous substitution, and numerous small SVs. Independent studies have identified major QTLs within the centromeric region of chromosome 5 associated with flowering time[63], hybrid yield[133], and up to 6 domestication traits in teosinte-maize backcross populations[134]. This region also overlaps with introgression intervals associated with adaptive variation in traditional maize varieties[32,69].

Large structural variants, such as inversions, can reinforce genetic barriers between populations by contributing to the fixation of deleterious or incompatible variants[135], and are frequently co-opted during drive evolution to ensure the integrity of distorter-responder pairs, and of linked bystander genes not involved in drive[11]. Functional analysis of variation in the *Tpd1* interval revealed a handful of putative loss-of-function mutations (n = 12) owing primarily to frameshifts, as well as high rates of nonsynonymous substitution (n = 277). This result was in line with the estimates of increased genetic load within regions of low recombination[136], and mirrored observations in other drive systems, which often accumulate deleterious recessive mutations in linkage[11]. In addition to coding sequence mutations, we found extensive structural variation in the form of 1519 high confidence insertions and deletions (indels) of various sizes (mean size = 5,419bp).

Teosinte pollen drive intervals that persist in backcrosses have *mexicana* ancestry in traditional varieties of maize

Hybridization between teosinte *mexicana* and early cultivated maize was a critical step in the adaptation and geographical dispersal of modern maize, following initial domestication from teosinte *parviglumis* in tropical lowlands[32,137,138]. We rationalized that, if *TPD* exists as a cryptic drive system in *mexicana*, signatures of drive might be found in traditional Mexican varieties (often called "landraces"[32]), and in sympatric *mexicana* populations in the central highlands of Mexico where hybridization still occurs. Approximately 13 intervals of the *mexicana* genome persisted in serial backcrosses to the maize inbred W22 (Extended Data Fig. 2a,b), and we looked for signatures of these intervals in 256 accessions from 10 different traditional varieties of maize, along with their sympatric populations of wild *mexicana*[32]. We determined the mean frequency of *mexicana*-like alleles from each interval in each population from existing genotyping data[138] and determined the Spearman's rank correlation coefficient for each pair of intervals. We found that, in *mexicana* populations, the intervals encoding *Tpd1* (chr 5.79), *Tpd2* (chr 6.98) and *Rgd1* (chr 6.3) were highly correlated (Extended Data Fig. 9a), suggesting that this combination of toxin and antidote loci is ancestral in these *mexicana* populations. In traditional maize varieties, on the other hand, significant correlations were observed between 11 of the 13 intervals (Extended Data Fig. 9a).

Components of Teosinte pollen drive have undergone selection during domestication

To further characterize the evolution of genomic elements associated with *TPD*, we applied the haplotype-based statistic iHS[139] and the F_{ST} statistic[140] to identify genome-wide signals of natural selection (see Methods) using variant data for *parviglumis*, *mexicana*, traditional varieties, and modern lines[120,121]. We considered values in the top 5% of the genome-wide empirical distributions of these statistics as significant. F_{ST} was effective at identifying known domestication loci, but did not show significant signals of selection for the *TPD*-linked genes (Supplementary Table 4). However, we found that *dcl2*, *tdr1*, *rdm1* and the hairpin region contained significant SNPs in teosinte based on iHS (Supplementary Table 5), indicating these loci may be under selection. More conservative analyses using Bonferroni *p*-value adjustment for the multiple tested populations and windowed iHS statistics found signatures of selection in *dcl2* for *mexicana* and for the hairpin region in *parviglumis* (Supplementary Table 5). Finally, the 13Mb inversion within the *Tpd1* haplotype encompasses one of largest known domestication sweeps between maize inbreds and teosinte[4], accounting perhaps for the unusual size and rapid emergence of "Region D". Signals of selection at *TPD* loci themselves, by contrast, were weaker than at known domestication loci, consistent with the idea that drive itself is not adaptive.

Supplementary tables for

Teosinte Pollen Drive guides maize domestication and evolution by RNAi.

Supplementary Table 1: Phenotypic Segregation Ratios for Reciprocal *TPD* Crosses

Cross	Fertile	Semi-Sterile	Sterile	Chi-square
TPD ♂	0	142	0	$\chi^2(2:1:1) = 164.29$, $p < 0.00001$
TPD ♂	0	179	0	$\chi^2(2:1:1) = 209.29$, $p < 0.00001$
TPD ♂	0	219	0	$\chi^2(2:1:1) = 257.27$, $p < 0.00001$
TPD ♀	144	89	58	$\chi^2(2:1:1) = 3.301$, $p = 0.192$
TPD ♀	159	109	71	$\chi^2(2:1:1) = 5.234$, $p = 0.073$
TPD ♀	106	60	54	$\chi^2(2:1:1) = 0.193$, $p = 0.908$

Supplementary Table 2: Genotypic Segregation Ratios for Reciprocal *TPD* Crosses

Parent	*tpd1; tpd2*	*Tpd2+/-*	*Tpd/++*	*Tpd1+/-*	Chi-square
TPD ♂	0	0	142	0	$\chi^2(1:1:1:1) = 162.44$, $p < 0.00001$
TPD ♂	0	0	179	0	$\chi^2(1:1:1:1) = 204.41$, $p < 0.00001$
TPD ♂	0	0	219	0	$\chi^2(1:1:1:1) = 254.37$, $p < 0.00001$
TPD ♀	81	63	89	58	$\chi^2(1:1:1:1) = 4.447$, $p = 0.217$
TPD ♀	69	90	109	71	$\chi^2(1:1:1:1) = 6.029$, $p = 0.110$
TPD ♀	51	55	60	54	$\chi^2(1:1:1:1) = 0.378$, $p = 0.945$

Supplementary Table 3: Genome Assembly Metrics. Values calculated on scaffolded assemblies with contigs split by 10 or more consecutive Ns. Repeat content determined by RepeatMasker with the combined MTEC repeat library "maizeTE02052020" distributed at https://github.com/oushujun/MTEC.

	**TPD**<br>**TPD 1.0 2021**	**W22**<br>**W22 Reference NRGENE-2.0 2018**
**Contigs**		
#	945	49,111
Longest (Kbp)	44,353	831
N50 (Kbp)	7,744	88
**Scaffolds**		
# > 10Mbp	10	10
Span (Mbp)	2,112	2,134
Longest (Mbp)	306	311
Gap bases (%)	0.02	1.90
**Annotations**		
Coding Genes (W22 liftover)	40,564	40,961
Interspersed Repeats (%)	81.37	80.04
CentC masked (Kbp)	2,876	137
**QC**		
merqury QV estimate	34.2	-
Complete BUSCOs (%)	90.6	91.1
Missing BUSCOs (%)	0.3	0.6

Supplementary Table 6: Empirical *p*-values for selection scan with a windowed weighted F_{ST} statistic for *Teosinte Pollen Drive*-linked (*TPD*-linked) regions and a validation set of domestication genes. See Supplementary Table 5 for gene coordinates. Empirical *p*-values for each gene are shown for the overlapping window with the lowest *p*-value. Significant *p*-values <0.05 are indicated with an asterisk for both unadjusted and Bonferroni-adjusted values accounting for each population pair tested.

		***mexicana* - Landrace**	***parviglumis* - Landrace**	**Landrace - Modern**	***mexicana* - Modern**	***parviglumis* - Modern**
**Group**	**Gene**	**Windowed F_{ST} empirical *p*-values**				
TPD-linked	*dcl2*	0.321	0.584	0.479	0.132	0.345
TPD-linked	hairpin	0.305	0.503	0.742	0.106	0.255
TPD-linked	*rdm1*	0.510	0.660	0.326	0.585	0.802
TPD-linked	*tdr1*	0.263	0.324	0.166	0.096	0.124
Domestication	*gt1*	0.128	0.058	0.020*	0.213	0.164
Domestication	*tb1*	0.016*	0.110	0.329	0.004*	0.047*
Domestication	*tga1*	0.032*	0.003*	0.356	0.021*	0.004*
Domestication	*zagl1*	0.025*	0.001*	0.201	0.003*	0.001*
		**Windowed F_{ST} empirical adjusted *p*-values**				
TPD-linked	*dcl2*	1.000	1.000	1.000	0.662	1.000
TPD-linked	hairpin	1.000	1.000	1.000	0.529	1.000
TPD-linked	*rdm1*	1.000	1.000	1.000	1.000	1.000
TPD-linked	*tdr1*	1.000	1.000	0.828	0.482	0.622
Domestication	*gt1*	0.638	0.290	0.100	1.000	0.822
Domestication	*tb1*	0.082	0.551	1.000	0.021*	0.236
Domestication	*tga1*	0.158	0.016*	1.000	0.105	0.020*
Domestication	*zagl1*	0.123	0.004*	1.000	0.013*	0.003*

Supplementary Table 7: Selection scan with a windowed |iHS| statistic in *Teosinte Pollen Drive*-linked (*TPD*-linked) regions and a validation set of domestication genes in teosinte and maize populations. Counts of individual significant (p<0.05) SNPs as well as empirical p-values for 10kb windows are shown. Genomic coordinates for *dcl2* (Zm00001eb219690), *tdr1* (Zm00001eb224090) and *rdm1* (Zm00001eb275620) are for the public maize B73 NAMv5 reference genome and annotation from MaizeGDB. Coordinates for the domestion genes are based on the same assembly and annotation. Significant p-values <0.05 are indicated with an asterisk. SNP counts and per window p-values are shown as unadjusted and Bonferroni-adjusted values accounting for each population tested. Cases in which there was insufficient data to calculate a p-value are shown as "NA".

Group	Gene	Genomic coordinates	***mexicana***	***parviglumis***	**Landrace**	**Modern**
			**Count of |iHS| significant SNPs**			
TPD-linked	*dcl2*	Chr5:20,831,070-20,848,118 (-)	46	6	1	18
TPD-linked	*tdr1*	Chr5:40,759,118-40,761,756 (-)	1	0	0	0
TPD-linked	hairpin	Chr5:95,990,889-96,002,706 (+)	4	30	1	0
TPD-linked	*rdm1*	Chr6:107,817,218-107,820,394 (+)	0	2	0	14
Domestication	*zagl1*	Chr5:20,831,070-20,848,118 (-)	48	40	0	0
Domestication	*gt1*	Chr5:40,759,118-40,761,756 (-)	0	0	0	3
Domestication	*tb1*	Chr5:95,990,889-96,002,706 (+)	5	2	2	0
Domestication	*tga1*	chr1:272330564-272332648 (+)	2	0	0	0
			**Count of |iHS| adjusted significant SNPs**			
TPD-linked	*dcl2*	Chr5:20,831,070-20,848,118 (-)	10	0	0	0
TPD-linked	*tdr1*	Chr5:40,759,118-40,761,756 (-)	0	0	0	0
TPD-linked	hairpin	Chr5:95,990,889-96,002,706 (+)	0	10	0	0
TPD-linked	*rdm1*	Chr6:107,817,218-107,820,394 (+)	0	0	0	6
Domestication	*zagl1*	Chr1:4932248-4948340 (-)	0	12	0	0
Domestication	*gt1*	chr1:23433554-23435122 (+)	0	0	0	3
Domestication	*tb1*	chr1:272330564-272332648 (+)	4	1	2	0
Domestication	*tga1*	chr4:46647932-46652896 (+)	0	0	0	0
			**Windowed |iHS| empirical *p*-values**			
TPD-linked	*dcl2*	Chr5:20,831,070-20,848,118 (-)	0.008*	0.341	0.316	0.079

TPD-linked	*tdr1*	Chr5:40,759,118-40,761,756 (-)	0.377	0.649	0.759	0.468
TPD-linked	hairpin	Chr5:95,990,889-96,002,706 (+)	0.216	0.171	0.493	0.215
TPD-linked	*rdm1*	Chr6:107,817,218-107,820,394 (+)	0.421	0.414	0.737	0.021*
Domestication	*zagl1*	Chr5:20,831,070-20,848,118 (-)	0.020*	0.039*	NA	NA
Domestication	*gt1*	Chr5:40,759,118-40,761,756 (-)	0.913	0.373	0.780	0.410
Domestication	*tb1*	Chr5:95,990,889-96,002,706 (+)	0.021*	0.116	0.054	0.188
Domestication	*tga1*	chr1:272330564-272332648 (+)	0.315	0.477	0.641	0.673
			**Windowed |iHS| empirical adjusted *p*-values**			
TPD-linked	*dcl2*	Chr5:20,831,070-20,848,118 (-)	0.031*	1.000	1.000	0.314
TPD-linked	*tdr1*	Chr5:40,759,118-40,761,756 (-)	1.000	1.000	1.000	1.000
TPD-linked	hairpin	Chr5:95,990,889-96,002,706 (+)	0.863	0.686	1.000	0.861
TPD-linked	*rdm1*	Chr6:107,817,218-107,820,394 (+)	1.000	1.000	1.000	0.085
Domestication	*zagl1*	Chr1:4932248-4948340 (-)	0.080	0.156	NA	NA
Domestication	*gt1*	chr1:23433554-23435122 (+)	1.000	1.000	1.000	1.000
Domestication	*tb1*	chr1:272330564-272332648 (+)	0.082	0.465	0.217	0.753
Domestication	*tga1*	chr4:46647932-46652896 (+)	1.000	1.000	1.000	1.000

Supplementary Table 8: Genotyping markers used in this study. The "type" column refers to the general marker design associated with the primer sequence. RFLP markers will denote the associated restriction enzyme in the "type" column.

Name	Type	Sequence (5'-3')	WT	Mut/*TPD*
dcl2^{T}f	BstNI	AGCGCCATTTACAATTTCAGCA	183, 211	394
dcl2^{T}-r	BstNI	TGTTGCCAGTGAATCAGCACTA	183, 211	394
m5.0-f	SSLP	TTAGTAGTGTCCTGGCGCTC	295	165
m5.0-r	SSLP	GTGAGGGACTAGGGCATGTG	295	165
m5.1-f	SSLP	CCTGCATAGAGATGCCATCAA	310	180
m5.1-r	SSLP	CGACGACGACTCATCCACGA	310	180
m5.2-f	SSLP	TGTCTTCCTCCAACTGTGCT	312	211
m5.2-r	SSLP	ACTGCCCAAAGAGCATGTGT	312	211
m5.3-f	MfeI	AATGGTGTTTTCCTTGGCATTCA	253, 249	502
m5.3-r	MfeI	GTACCATGCACTCATCCCGAA	253, 249	502
m5.4-f	TseI	GGATCATGGAGTGCGTGCAG	450	161, 289
m5.4-r	TseI	AACCAGCGCTCCTCAAAGTT	450	161, 289
m6.0-f	SSLP	ACTGAGTAACCAATGCCAGA	445	363
m6.0-r	SSLP	GCAGCCTTCCAGTTCCTGTA	445	363
m6.1-f	SSLP	GATCTACTTGCACGAGAGCACC	495	374
m6.1-r	SSLP	CGGAGTAATTTCCTTGGGACA	495	374
m6.2-f	AflIII	CTACCAACTGCTCCTGAGATGG	116, 96	212
m6.2-r	AflIII	CGTTGACGAATATTGATTGTAGCCA	116, 96	212
m6.3-f	NdeI	TTGCTCCAACCTTGTCACCT	124, 100	224
m6.3-r	NdeI	GCTATCCGCAAACAGCGAGA	124, 100	224
m6.4-f	BccI	TCCTTCTCCTCTTCCCTCGG	250	110, 140
m6.4-r	BccI	AGGAACCCTGTTTGACGATCT	250	110, 140
m6.5-f	BglIII	TCCACAGAAGGACAGCAAAAGGA	174, 65	239
m6.5-r	BglIII	TAGGGTTTGTTGTGGCTGCT	174, 65	239
m6.6-f	PvuII	GGCCAAGTTGTTCAAGAAGCAT	165, 85	254
m6.6-r	PvuII	GCGTGGCCCCTTCTCTTATT	165, 85	254
m6.7-f	NdeI	GGGCATCGTGTCATTGAAGG	231	115, 116
m6.7-r	NdeI	TGCAACCTCTCAGGTCTAAG	231	115, 116
lbl-rgd1-f	MwoI	GCCCATCTGGATCTGAAGTC	262, 227	489
lbl-rgd1-r	MwoI	TTGGTGGCCACACTATCTCA	262, 227	489
dcl2mu1-f	Mu insertion	GTGTCCGCGTTCCAGAAGTC	906	800
dcl2mu1-r	Mu insertion	TAAAGGTTGTCCATTGGGCGTT	906	800
TIR4	TIR	GCCTCCATTTCGTCGAATCCC	-	-
TIR6	TIR	GCCTCTATTTCGTCGAATCCG	-	-
Gdsl-f	HiII check	GAGCGTCTCCTTCAACCCAA	983	-
Gdsl-r	HiII check	TCCTACTCCTCAGTTGGGGG	983	-
Dcl2-f	HiII check	GGCCTAGAATTCAGTTGCGG	717	-
Dcl2-r	HiII check	GAACACGCTTGTGTTCCTCG	717	-

Supplementary Table 9: RT-qPCR primers used in this study. All RT-qPCR primers used in this study were designed to bridge exon junctions (if present) in order to increase specificity. Expected amplicon sizes corresponding to a cDNA or gDNA template are listed.

Name	Target	Sequence	cDNA	gDNA
Dcl2q-1f	*Dcl2* – exon 1,2	CCCAAAAGGACACACAGCTTTC	79	159
Dcl2q-1r	*Dcl2* – exon 1,2	GCATATCTGATCTTGGAGTATGGC	79	159
Dcl2q-2f	*Dcl2* – exon 5,6	TCGACAAAAACAATGCATCTCAAAT	104	658
Dcl2q-2r	*Dcl2* – exon 5,6	GCTGAGGATCTGCAAGATGG	104	658
Dcl2q-3f	*Dcl2* – exon 18,19	CATTCTGAAGGGTGTCGGGT	174	417
Dcl2q-3r	*Dcl2* – exon 18,19	GGTCCATCACATGGTTGGGAA	174	417
Gdslq-1f	*Gdsl* – exon 1,2	GAGCGTCTCCTTCAACCCAA	81	249
Gdslq-1r	*Gdsl* – exon 1,2	ACAAGGCTACTGGCAGGTTC	81	249
Gdslq-2f	*Gdsl* – exon 2,3	CCCACCTTCGCCTTGTACTC	153	250
Gdslq-2r	*Gdsl* – exon 2,3	CCTGAACAAGAGCCTCACCC	153	250
Elfa9q-1f	*Efla9* – exon 1,2	CAAGCTGACTGTGCTGTTCTTA	181	928
Elfa9q-1r	*Efla9* – exon 1,2	GCCTTTGAATACTTGGGTGTAGTAG	181	928
Elfa9q-2f	*Efla9* – exon 1,2	CCAAGCTGACTGTGCTGTTCTT	198	945
Elfa9q-2r	*Efla9* – exon 1,2	AATCTCATCATAACGGGCCTTTGAA	198	945
Rdr6q-1f	*Rdr6* – no exons	TGTTTTGCTTCAGCTGGGGA	128	128
Rdr6q-1r	*Rdr6* – no exons	CACGCGAATGAAGCATCTGG	128	128
Rdr6q-2f	*Rdr6* – no exons	GCCTCTCACCTTTCTTGGGG	74	74
Rdr6q-2r	*Rdr6* – no exons	CCTTGAAGCTGTTGATGTGCC	74	74
Rgd1q-1f	*Rgd1* – exon 5,6	GCCACATCCTTGACTTGGCA	119	319
Rgd1q-1r	*Rgd1* – exon 5,6	TGCAGGAAGAGCGCTCAAAG	119	319
Rgd1q-2f	*Rgd1* – exon 4,6	CGCTCTTCCTGCAACAACTT	130	378
Rgd1q-2r	*Rgd1* – exon 4,6	GAGAAGCACATGGAGTACGAGG	130	378
Ago1e-1f	*Ago1e* – exon 19,20	TTCTACCTGTGCAGCCATGC	103	172
Ago1e-1r	*Ago1e* – exon 19,20	GAGTTTGCAACCCATCAGCC	103	172
Ago1e-1f	*Ago1e* – exon 10,11	GCCAAAAATTGGCCAGTGGA	128	515
Ago1e-1r	*Ago1e* – exon 10,11	TCATGACAGAAACTCCCGGC	128	515

129. Haig, D. & Grafen, A. Genetic scrambling as a defence against meiotic drive. *J. Theor. Biol.* **153**, 531–558 (1991).
130. Hurst, G. D. & Werren, J. H. The role of selfish genetic elements in eukaryotic evolution. *Nat. Rev. Genet.* **2**, 597–606 (2001).
131. Johnson, N. A. Hybrid incompatibility genes: remnants of a genomic battlefield? *Trends Genet.* **26**, 317–325 (2010).
132. Lyon, M. F. Transmission ratio distortion in mouse t-haplotypes is due to multiple distorter genes acting on a responder locus. *Cell* **37**, 621–628 (1984).
133. Stuber, C. W., Lincoln, S. E., Wolff, D. W., Helentjaris, T. & Lander, E. S. Identification of genetic factors contributing to heterosis in a hybrid from two elite maize inbred lines using molecular markers. *Genetics* **132**, 823–839 (1992).
134. Briggs, W. H., McMullen, M. D., Gaut, B. S. & Doebley, J. Linkage mapping of domestication loci in a large maize teosinte backcross resource. *Genetics* **177**, 1915–1928 (2007).
135. Ayala, F. J. & Coluzzi, M. Chromosome speciation: humans, Drosophila, and mosquitoes. *Proc. Natl. Acad. Sci. U. S. A.* **102 Suppl 1**, 6535–6542 (2005).
136. Rodgers-Melnick, E. *et al.* Recombination in diverse maize is stable, predictable, and associated with genetic load. *Proc. Natl. Acad. Sci. U. S. A.* **112**, 3823–3828 (2015).
137. McLean-Rodríguez, F. D., Costich, D. E., Camacho-Villa, T. C., Pè, M. E. & Dell'Acqua, M. Genetic diversity and selection signatures in maize landraces compared across 50 years of in situ and ex situ conservation. *Heredity* **126**, 913–928 (2021).
138. Calfee, E. *et al.* Selective sorting of ancestral introgression in maize and teosinte along an elevational cline. *PLoS Genet.* **17**, e1009810 (2021).
139. Voight, B. F., Kudaravalli, S., Wen, X. & Pritchard, J. K. A map of recent positive selection in the human genome. *PLoS Biol.* **4**, e72 (2006).
140. Weir, B. S. & Cockerham, C. C. ESTIMATING F-STATISTICS FOR THE ANALYSIS OF POPULATION STRUCTURE. *Evolution* **38**, 1358–1370 (1984)
