# Supplementary material for: Teosinte Pollen Drive guides maize diversification and domestication by RNAi
Source: Nature. 2024 Aug 7;633(8029):380–8. doi: 10.1038/s41586-024-07788-0 (PMC11390486; doi:10.1038/s41586-024-07788-0)
Supplement: Supplementary file 2 — Reporting Summary [file 41586_2024_7788_MOESM2_ESM.pdf]

nature portfolio

Corresponding author(s): Robert Martienssen

Last updated by author(s): Jun 18, 2024

Reporting Summary

Nature Portfolio wishes to improve the reproducibility of the work that we publish. This form provides structure for consistency and transparency in reporting. For further information on Nature Portfolio policies, see our Editorial Policies and the Editorial Policy Checklist.

Statistics

For all statistical analyses, confirm that the following items are present in the figure legend, table legend, main text, or Methods section.

n/a	Confirmed	
☐	☒	The exact sample size (*n*) for each experimental group/condition, given as a discrete number and unit of measurement
☐	☒	A statement on whether measurements were taken from distinct samples or whether the same sample was measured repeatedly
☐	☒	The statistical test(s) used AND whether they are one- or two-sided<br>*Only common tests should be described solely by name; describe more complex techniques in the Methods section.*
☐	☒	A description of all covariates tested
☐	☒	A description of any assumptions or corrections, such as tests of normality and adjustment for multiple comparisons
☐	☒	A full description of the statistical parameters including central tendency (e.g. means) or other basic estimates (e.g. regression coefficient) AND variation (e.g. standard deviation) or associated estimates of uncertainty (e.g. confidence intervals)
☐	☒	For null hypothesis testing, the test statistic (e.g. *F*, *t*, *r*) with confidence intervals, effect sizes, degrees of freedom and *P* value noted<br>*Give P values as exact values whenever suitable.*
☐	☒	For Bayesian analysis, information on the choice of priors and Markov chain Monte Carlo settings
☐	☒	For hierarchical and complex designs, identification of the appropriate level for tests and full reporting of outcomes
☐	☒	Estimates of effect sizes (e.g. Cohen's *d*, Pearson's *r*), indicating how they were calculated

Our web collection on statistics for biologists contains articles on many of the points above.

Software and code

Policy information about availability of computer code

Data collection	No software was used for data collection.
Data analysis	All analysis are described in the Methods section of the manuscript. All code is available on Github (https://github.com/martienssenlab/TPD-manuscript). The following version of the softwares have been used: Cutadapt v3.1; BWA-MEM v0.7.17; Samtools v1.10; FreeBayes v1.3.2; Guppy v5.0.7; Flye v2.9-b1768; Minimap2 v2.22-r1101; PEPPER-Margin-DeepVariant v0.4; bwa-mem2 v2.2.1; NextPolish v1.3.1; Hapo-G v1.2; Juicer v1.6; 3D-DNA; JBAT; Liftoff 1.6.2; BCFtools v1.14; GLIMPSE v1.1.1; STAR v2.7.5c; featureCounts v2.0.1; edgeR v3.32.1; topGO v2.42.0; rrvgo v1.5.3; deepTools v3.5.0; bowtie2 v2.4.1; Shortstack v3.8.5; RNAfold; BEDtools v2.30; psRNATarget; selscan v1.2.0a; VCFTools v0.1.16; Beagle v5.4;

For manuscripts utilizing custom algorithms or software that are central to the research but not yet described in published literature, software must be made available to editors and reviewers. We strongly encourage code deposition in a community repository (e.g. GitHub). See the Nature Portfolio guidelines for submitting code & software for further information.

Data

Policy information about availability of data

All manuscripts must include a data availability statement. This statement should provide the following information, where applicable:

- Accession codes, unique identifiers, or web links for publicly available datasets
- A description of any restrictions on data availability
- For clinical datasets or third party data, please ensure that the statement adheres to our policy

Sequencing datasets generated during the current study are available at NCBI (GEO SuperSeries: GSE234925) and datasets used for genome assembly are available at SRA (BioProject: PRJNA937229).

Research involving human participants, their data, or biological material

Policy information about studies with human participants or human data. See also policy information about sex, gender (identity/presentation), and sexual orientation and race, ethnicity and racism.

Reporting on sex and gender	NA
Reporting on race, ethnicity, or other socially relevant groupings	NA
Population characteristics	NA
Recruitment	NA
Ethics oversight	NA

Note that full information on the approval of the study protocol must also be provided in the manuscript.

Field-specific reporting

Please select the one below that is the best fit for your research. If you are not sure, read the appropriate sections before making your selection.

☒ Life sciences ☐ Behavioural & social sciences ☐ Ecological, evolutionary & environmental sciences

For a reference copy of the document with all sections, see nature.com/documents/nr-reporting-summary-flat.pdf

Life sciences study design

All studies must disclose on these points even when the disclosure is negative.

Sample size	No sample-size calculations were performed for this study. Sample sizes were chosen to match or exceed standards in the field.
Data exclusions	No data was excluded from the analysis.
Replication	At least 2 biological replicates were performed for all experiments, except for genome assembly. The number of replicates is indicated for each experiment. All attempts at replication were successful.
Randomization	Plants were grown in random order in the field and greenhouse, and were phenotyped in the same random order.
Blinding	Phenotyping and pollen grain viability staining experiments were performed blindly, without previous knowledge of the genotype.

Reporting for specific materials, systems and methods

We require information from authors about some types of materials, experimental systems and methods used in many studies. Here, indicate whether each material, system or method listed is relevant to your study. If you are not sure if a list item applies to your research, read the appropriate section before selecting a response.

Materials & experimental systems

n/a	Involved in the study	
☐	☒	Antibodies
☒	☐	Eukaryotic cell lines
☒	☐	Palaeontology and archaeology
☒	☐	Animals and other organisms
☒	☐	Clinical data
☒	☐	Dual use research of concern
☐	☒	Plants

Methods

n/a	Involved in the study	
☒	☐	ChIP-seq
☒	☐	Flow cytometry
☒	☐	MRI-based neuroimaging

Antibodies

Antibodies used	anti-HSP90-2 (Agrisera, cat#AS11 1629)<br>antiserum raised against a peptide (SRKGAPPSSPPLSPPKLGA) from the Zm00004b012122 protein in collaboration with PhytoAB
Validation	AS11 1629 - manufacturer tested for Western blot.<br>Specificity of antiserum was determined as follows: (1) blots using pollen protein extracts showed a single band at roughly the expected size, and (2) blots using leaf protein extracts showed no band in concordance with expected pollen/anther specificity

Dual use research of concern

Policy information about dual use research of concern

Hazards

Could the accidental, deliberate or reckless misuse of agents or technologies generated in the work, or the application of information presented in the manuscript, pose a threat to:

No	Yes	
☒	☐	Public health
☒	☐	National security
☒	☐	Crops and/or livestock
☒	☐	Ecosystems
☒	☐	Any other significant area

Experiments of concern

Does the work involve any of these experiments of concern:

No	Yes	
☒	☐	Demonstrate how to render a vaccine ineffective
☒	☐	Confer resistance to therapeutically useful antibiotics or antiviral agents
☒	☐	Enhance the virulence of a pathogen or render a nonpathogen virulent
☒	☐	Increase transmissibility of a pathogen
☒	☐	Alter the host range of a pathogen
☒	☐	Enable evasion of diagnostic/detection modalities
☒	☐	Enable the weaponization of a biological agent or toxin
☒	☐	Any other potentially harmful combination of experiments and agents

Plants

Seed stocks	See Methods for origin of TPD material. dcl2-mu1 was isolated from Uniform-Mu line UFMu-12288. lbl1-rgd1 was ordered from the Maize Genetics Cooperation Stock Center (601C).
Novel plant genotypes	Mutations in Tdr1 were performed by CRISPR-Cas9 at the Plant Transformation Core Facility at the University of Missouri.
Authentication	Genotypes were assessed by PCR and confirmed by Sanger sequencing, as detailed in the Methods and with primers listed in supplementary tables 8 and 9.
